# Enhanced RNAi does not provide efficient innate antiviral immunity in mice

**DOI:** 10.1093/nar/gkae1288

**Published:** 2025-01-09

**Authors:** Marcos Iuri Roos Kulmann, Eliska Taborska, Brigita Benköova, Martin Palus, Ales Drobek, Filip Horvat, Josef Pasulka, Radek Malik, Eva Salyova, Vaclav Hönig, Michaela Pellerova, Maria Borsanyiova, Lenka Nedvedova, Ondrej Stepanek, Shubhada Bopegamage, Daniel Ruzek, Petr Svoboda

**Affiliations:** Laboratory of Epigenetic Regulations, Institute of Molecular Genetics of the Czech Academy of Sciences, Videnska 1083, 142 20, Prague, Czech Republic; Laboratory of Epigenetic Regulations, Institute of Molecular Genetics of the Czech Academy of Sciences, Videnska 1083, 142 20, Prague, Czech Republic; Faculty of Medicine, Enterovirus Laboratory, Institute of Microbiology, Slovak Medical University, Limbova 12, 83303Bratislava, Slovakia; Institute of Parasitology, Biology Centre of the Czech Academy of Sciences, Branisovska 31, CZ-37005 Ceske Budejovice, Czech Republic; Laboratory of Emerging Viral Infections, Veterinary Research Institute, Hudcova 70, CZ-62100 Brno, Czech Republic; Laboratory of Adaptive Immunity, Institute of Molecular Genetics of the Czech Academy of Sciences, Videnska 1083, 142 20, Prague, Czech Republic; Laboratory of Epigenetic Regulations, Institute of Molecular Genetics of the Czech Academy of Sciences, Videnska 1083, 142 20, Prague, Czech Republic; Bioinformatics Group, Department of Biology, Faculty of Science, University of Zagreb, Horvatovac 102a, 10000 Zagreb, Croatia; Laboratory of Epigenetic Regulations, Institute of Molecular Genetics of the Czech Academy of Sciences, Videnska 1083, 142 20, Prague, Czech Republic; Laboratory of Epigenetic Regulations, Institute of Molecular Genetics of the Czech Academy of Sciences, Videnska 1083, 142 20, Prague, Czech Republic; Laboratory of Adaptive Immunity, Institute of Molecular Genetics of the Czech Academy of Sciences, Videnska 1083, 142 20, Prague, Czech Republic; Institute of Parasitology, Biology Centre of the Czech Academy of Sciences, Branisovska 31, CZ-37005 Ceske Budejovice, Czech Republic; Laboratory of Emerging Viral Infections, Veterinary Research Institute, Hudcova 70, CZ-62100 Brno, Czech Republic; Faculty of Medicine, Enterovirus Laboratory, Institute of Microbiology, Slovak Medical University, Limbova 12, 83303Bratislava, Slovakia; Faculty of Medicine, Enterovirus Laboratory, Institute of Microbiology, Slovak Medical University, Limbova 12, 83303Bratislava, Slovakia; Institute of Parasitology, Biology Centre of the Czech Academy of Sciences, Branisovska 31, CZ-37005 Ceske Budejovice, Czech Republic; Faculty of Science, University of South Bohemia, Branisovska 1645/31a, CZ-37005Ceske Budejovice, Czech Republic; Laboratory of Adaptive Immunity, Institute of Molecular Genetics of the Czech Academy of Sciences, Videnska 1083, 142 20, Prague, Czech Republic; Faculty of Medicine, Enterovirus Laboratory, Institute of Microbiology, Slovak Medical University, Limbova 12, 83303Bratislava, Slovakia; Institute of Parasitology, Biology Centre of the Czech Academy of Sciences, Branisovska 31, CZ-37005 Ceske Budejovice, Czech Republic; Laboratory of Emerging Viral Infections, Veterinary Research Institute, Hudcova 70, CZ-62100 Brno, Czech Republic; Department of Experimental Biology, Faculty of Science, Masaryk University, Kamenice 5, CZ-62500Brno, Czech Republic; Laboratory of Epigenetic Regulations, Institute of Molecular Genetics of the Czech Academy of Sciences, Videnska 1083, 142 20, Prague, Czech Republic

## Abstract

In RNA interference (RNAi), long double-stranded RNA is cleaved by the Dicer endonuclease into small interfering RNAs (siRNAs), which guide degradation of complementary RNAs. While RNAi mediates antiviral innate immunity in plants and many invertebrates, vertebrates have adopted a sequence-independent response and their Dicer produces siRNAs inefficiently because it is adapted to process small hairpin microRNA precursors in the gene-regulating microRNA pathway. Mammalian endogenous RNAi is thus a rudimentary pathway of unclear significance. To investigate its antiviral potential, we modified the mouse Dicer locus to express a truncated variant (*Dicer*^ΔHEL1^) known to stimulate RNAi and we analyzed how *Dicer*^ΔHEL1/wt^ mice respond to four RNA viruses: coxsackievirus B3 and encephalomyocarditis virus from *Picornaviridae*; tick-borne encephalitis virus from *Flaviviridae*; and lymphocytic choriomeningitis virus (LCMV) from *Arenaviridae*. Increased Dicer activity in *Dicer*^ΔHEL1/wt^ mice did not elicit any antiviral effect, supporting an insignificant antiviral function of endogenous mammalian RNAi *in vivo*. However, we also observed that sufficiently high expression of Dicer^ΔHEL1^ suppressed LCMV in embryonic stem cells and in a transgenic mouse model. Altogether, mice with increased Dicer activity offer a new benchmark for identifying and studying viruses susceptible to mammalian RNAi *in vivo*.

## Graphical Abstract

**Graphical Abstract:**
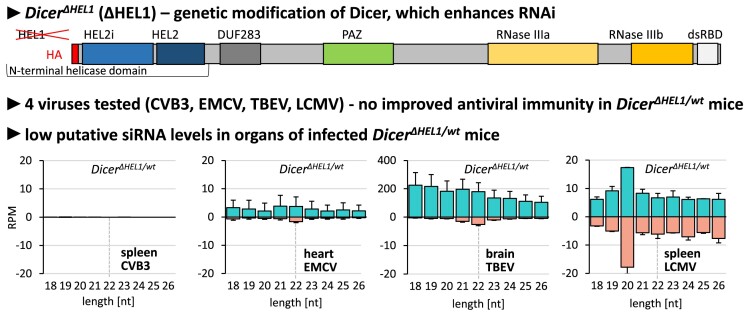

## Introduction

RNA interference (RNAi) means sequence-specific RNA degradation induced by long double-stranded RNA (dsRNA) (1). RNAi starts with RNase III Dicer cutting long dsRNA into 21–23-nt long small interfering RNAs (siRNAs), which are loaded onto Argonaute endonucleases to form the RNA-induced Silencing Complex (RISC), in which they serve as guides for recognition and cleavage of complementary RNAs. RNAi acquired many biological roles including gene regulation, antiviral immunity or defense against mobile elements [reviewed in (2)]. At the same time, vertebrate evolution brought curtailed RNAi and adaptation of Dicer to biogenesis of gene-regulating microRNAs (miRNAs) [reviewed in (3)], which are small RNAs released by Dicer from genome-encoded small hairpin precursors.

The key feature of Dicer differentiating RNAi and/or miRNA pathway support is its N-terminal helicase domain, which can mediate recognition and ATP-dependent processive cleavage of long dsRNA and/or promote ATP-independent pre-miRNA recognition and processing [reviewed in (4)]. During vertebrate evolution, the N-terminal helicase domain lost the ATPase activity and evolved to support high-fidelity processing of pre-miRNA (5–7). In fact, adaptation of mammalian Dicer for miRNA biogenesis made the N-terminal helicase an autoinhibitory element of long dsRNA processing (8). Consequently, the dominant class of Dicer products in almost all investigated mammalian cells is miRNA. Mammals retain a residual canonical RNAi response, i.e. mammalian Dicer is still able to cleave dsRNA into siRNAs, which are loaded onto AGO2 capable of mediating sequence-specific endonucleolytic cleavage of perfectly complementary targets (9–11). However, canonical RNAi in most mammalian cells is inefficient, in part owing to inefficient processing of long dsRNA into siRNAs (5,8,12,13) and in part to other long dsRNA responses in mammalian cells (including adenosine deamination and the interferon response), which hamper RNAi (13–16). An exception of the rule is the mouse oocyte, where functionally important endogenous RNAi emerged through an oocyte-specific promoter of retrotransposon origin, which expresses a truncated Dicer variant (denoted as Dicer^O^). Dicer^O^ lacks the HEL1 subdomain of the N-terminal helicase, efficiently cleaves long dsRNA and supports functionally relevant canonical RNAi (13,17).

RNAi can provide antiviral innate immunity. It was first shown for the flock house virus (18) and the vesicular stomatitis virus (VSV) (19,20), viruses of a broad host range, and later with natural viruses of *Caenorhabditis elegans* (21,22) and *Drosophila melanogaster* (23,24). The situation in mammals is complicated. In 2013, two reports provided evidence suggesting that RNAi may have an antiviral role in mammals (25,26). Following these initial findings on Nodamura virus and encephalomyocarditis virus (EMCV) that were immediately intensely debated (27), several other studies reported functional antiviral RNAi against the influenza A virus (IAV) (28), human enterovirus 71 (29–31), Zika virus (ZIKV) (32,33), dengue virus type 2 (DENV2) (34,35), Semliki forest virus (SFV) (31) and Sindbis virus (SINV) (31,33). Showing effects in mice and/or cultured cells, these studies argued that (i) viral siRNAs (vsiRNAs) are produced in infected cells and suppress viruses, (ii) antiviral effects are most visible in interferon weakened/deficient hosts and (iii) viruses have evolved viral suppressor of RNAi proteins counteracting RNAi (26,30,34,36). At the same time, other studies showed negligible levels or absent vsiRNAs and no antiviral effects of RNAi for a number of tested viruses including IAV (37), DENV2 (37,38), SINV (37,39–41), hepatitis C virus (38), West Nile virus (37,38), yellow fever virus (37,41), poliovirus (38), Venezuelan equine encephalitis virus (37), coxsackievirus B3 (CVB3) (41,42), VSV (31,37,38), measles virus (37), herpes simplex virus type 1 (37), severe acute respiratory syndrome coronavirus 2 (SARS-CoV-2) (31) and reovirus (37).

**Figure 1.**
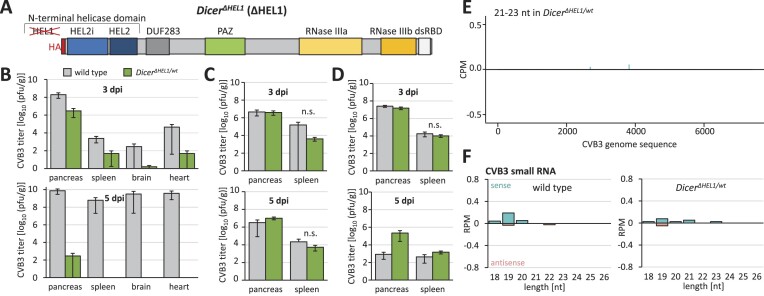
Infection of *Dicer^Δ HEL1/wt^* with CVB3 (*Picornaviridae*). (**A**) Schematic depiction of the Dicer^ΔHEL1^ protein isoform expressed from a modified endogenous Dicer locus. (B–D) Viral titers [plaque-forming unit (PFU) per g of tissue] at 3 and 5 dpi in different infection experiments. (**B**) Viral titers at 3 and 5 dpi in the pilot experiment, in which 4-week-old wild type mice developed systemic infection at 5 dpi but *Dicer*^*Δ*^*^HEL1/wt^* mice did not; *n* = 5 animals per genotype/timepoint. (**C**) Combined results of the next two infection experiments attempting to reproduce data from panel (B). Seven and twelve animals were used at 3 dpi for wild type and *Dicer*^*Δ*^*^HEL1/wt^* mice, respectively, five animals per genotype were used at 5 dpi. n.s. = statistically not significant reduction of viral titers in *Dicer*^*Δ*^*^HEL1/wt^* mice (one-tailed *t*-test). (**D**) Viral titers at 3 and 5 dpi after infection of 3-week-old mice (*n* = 5 animals per genotype/timepoint). (**E**) A coverage plot for CVB3 21–23-nt small RNAs derived from the + and − strand (displayed above and below the central line, respectively). The *x*-axis represents the viral genome sequence, the *y*-axis represents counts per million of 18–32-nt reads from two different infected spleens 3 dpi from 4-week-old mice analyzed in panel (C). (**F**) Distribution of frequencies of small RNAs of different lengths mapping onto the CVB3 genome in infected spleens. Error bars = SD. RPM = reads per million of 18–32-nt reads.

To obtain new insights into the antiviral potential of mammalian RNAi *in vivo*, we investigated viral resistance of mice in which RNAi activity was increased by expressing a Dicer^O^-like variant (6,43). We aimed at increasing siRNA production from long dsRNA while having a minimal effect on the miRNA pathway. In the main genetic model, we modified the endogenous Dicer locus to produce an N-terminally truncated Dicer variant (Dicer^ΔHEL1^, Figure 1A), which is functionally equivalent to naturally occurring Dicer^O^ (6). Although *Dicer^Δ HEL1/^*^ΔHEL1^ mice die perinatally from developmental defects, *Dicer*^Δ HEL1/wt^ mice are viable and fertile (6). Importantly, converting a half of endogenous Dicer expression into the highly active Dicer variant *in vivo* has a negligible effect on canonical miRNAs but brings an order of magnitude more siRNAs and several mirtrons (a non-canonical miRNA class) across organs (43). In our secondary RNAi model, Dicer^O^ was expressed from a transgene inserted into the ROSA26 locus (44), which provided higher Dicer^O^ expression and increased RNAi in different organs (44).

For infection, four RNA viruses, previously researched as mammalian pathogens in mice, were available to us. There were two viruses from the family *Picornaviridae*: CVB3 (strain Nancy) and EMCV, positive single-stranded RNA (ssRNA) viruses lacking an envelope. Their targeting by RNAi had been examined in cell culture previously. CVB3 was neither processed by full-length Dicer into vsiRNAs nor targeted by RNAi in AGO2-dependent manner in cultured cells (41,42), while EMCV was reported to be targeted by RNAi in embryonic stem cells (ESCs) (26). The next one was the tick-borne encephalitis virus (TBEV), a positive single-stranded enveloped virus from the family *Flaviviridae*, which is targeted by RNAi in the tick host (45). The last virus was the lymphocytic choriomeningitis virus (LCMV) from the family *Arenaviridae*, a family of segmented negative ssRNA enveloped viruses, whose targeting by endogenous mammalian RNAi had not been studied but was sensitive to targeting by exogenous siRNAs (46).

## Materials and methods

### Biological resources

#### Animals

Animal experiments were carried out in accordance with the Czech law and were approved by the Institutional Animal Use and Care Committee (approval nos. 34–2014, 29–2019 and 86–2020).

*Dicer^Δ HEL1^* mice were produced as previously described (6). For viral infection experiments, we used viable heterozygotes that were characterized in detail previously (43). *Pkr (Eif2ak2)* mutant mice and *Tg(PFGE-GAC-Dicer^O-HA^-mCHERRY)* transgenic mice on C57Bl/6 background were described elsewhere (43,44). Animals were genotyped by polymerase chain reaction (PCR). Briefly, tail biopsies were lysed in DEP-25 DNA Extraction buffer (Top-Bio) according to manufacturer’s instructions. One microliter aliquots was mixed with primers and Combi PPP Master Mix (Top-Bio) for the genotyping PCR. Genotyping primers are provided in Supplementary Table S1. For analyses, organs collected from sacrificed animals were either directly used for analysis or stored at −80°C for later use.

#### Cell lines

Mouse ESCs were cultured in 2i-LIF media: KnockOut™ DMEM (Gibco) supplemented with 15% fetal calf serum (Sigma–Aldrich), 1× L-glutamine (Thermo Fisher Scientific), 1× non-essential amino acids (Thermo Fisher Scientific), 50 μM β-mercaptoethanol (Gibco), 1000 U/ml LIF (Isokine), 1 μM PD0325901, 3 μM CHIR99021 (Selleck Chemicals), penicillin (100 U/ml) and streptomycin (100 μg/ml) at 37°C in a humidified atmosphere with 5% CO_2_.

Human lung adenocarcinoma A549 cells (ECACC 86012804) and mouse fibroblasts L929 were cultured in Dulbecco’s Modified Eagle’s Medium (DMEM) containing 10% fetal bovine serum (FBS; Biosera), 1% penicillin, 1% streptomycin and 1% glutamine (Biowest) at 37°C in a humidified atmosphere with 5% CO_2_.

Hamster BHK-21 and mouse 3T3 cells were cultured in DMEM containing 10% FBS (Biosera), 1% penicillin and 1% streptomycin at 37°C in a humidified atmosphere with 5% CO_2_.

#### Viral infections

##### CVB3

*In vivo* infections were conducted on the original CD1 genetic background of the *Dicer^Δ HEL1/wt^* mice in collaboration with the Enterovirus Laboratory at the Slovak Medical University. The virus was propagated in Vero cells (origin Public Health Institute, Bratislava), and 0.5 ml of 10^5^ TCID_50_/1ml virus per mouse was injected intraperitoneally. Mice were sacrificed and samples collected 3 and 5 days post infection (dpi); in two experiments, organs were also collected 7, 11 and 45 dpi.

##### EMCV

The EMCV isolate BCCO_50_0517 (GenBank: PP841942.1) was passaged on VERO/E6 prior to infection. This virus was provided by the Collection of Arboviruses, Biology Centre of the Czech Academy of Sciences (https://arboviruscollection.bcco.cz). *Dicer^Δ HEL1/wt^* mice on the C57Bl/6 background were infected subcutaneously into the scruff of the neck with 10^3^ PFUs. Mice were euthanized by cervical dislocation and heart samples were collected 2 and 3 dpi. The samples (2–5 mm^3^ of the organ) were placed into 600 μl Trizol and frozen (−80°C) for later RNA isolation. The identical EMCV stock was used for infection of mouse ESCs in multiplicity of infection (MOI) 1.0, supernatants were collected 24 or 72 hours post infection (hpi) and stored frozen (−80°C) for plaque titration.

##### TBEV

The TBEV strain Hypr (GenBank MT228627.1) was passaged five times in the brains of suckling mice before it was used in this study. This strain was provided by the Collection of Arboviruses, Biology Center of the Czech Academy of Sciences (https://arboviruscollection.bcco.cz). *Dicer^Δ HEL1/wt^* and *Dicer^Δ HEL1/wt^ Pkr^–/–^* mice on the C57Bl/6 background were infected subcutaneously into the scruff of the neck with 10^3^ PFUs. Mice were observed daily for body weight and clinical score (healthy, piloerection, hunched back, one leg paralyzed, two legs paralyzed, moribund and dead) as described previously (47) until 10 dpi. Mice were euthanized by cervical dislocation and brain samples were collected and homogenized as described previously (48). Brains were weighed individually and then homogenized in sterile phosphate buffered saline (PBS) (1:1) using the TissueLyser II (Qiagen). A total of 100 μl of the brain suspension was transferred to 600 μl Trizol and frozen (−80°C) for subsequent RNA isolation. The remaining homogenate was clarified by centrifugation at 14 000 × *g* for 10 min at 4°C and then used for virus titration using a plaque assay on A549 cells.

##### LCMV

Viral stocks of LCMV, strains Armstrong (Arm) and Clone 13 (C13), were propagated in BHK-21 cells as described previously (49). These strains were obtained from Prof. Daniel Pinschewer (University Hospital Basel, Switzerland). The viral titer in aliquots was determined by LCMV Focus Forming Assay. Briefly, 3T3 cells were infected with different dilutions of the virus supernatant. Viral antigens were detected with rat anti-LCMV nucleoprotein antibody (Clone VL-4; BioXCell, Cat. No.: BE0106, Lot: 787521S1, diluted 1:500) and visualized by a color reaction using secondary goat anti-rat IgG Horseradish peroxidase (HRP) antibody (diluted 1:500) 48 hpi. For LCMV acute infection, each mouse was injected intraperitoneally with 2 × 10^5^ or 2.5 × 10^6^ PFUs of LCMV Arm. At 3 or 8 dpi, mice were euthanized by cervical dislocation and organ samples were collected for total RNA isolation. All used animals had the C57Bl/6 background. For LCMV chronic infection, each mouse was injected intravenously with 10^6^ PFUs of LCMV C13. At 30 dpi, mice were euthanized by cervical dislocation and organ samples were collected for total RNA isolation. Mouse ESCs were infected with Arm at MOI of 0.01 or 1.0 and collected 24 or 48 hpi.

### Tissue histology

After formalin fixation, tissues were dehydrated using graded alcohols, cleared with xylene, and infiltrated with paraffin wax. Appropriate small tissue pieces were collected in 4% formaldehyde and embedded in paraffin wax cassettes. The tissues from paraffin-embedded blocks were cut into 4–7-μm thick slices on a microtome and mounted from warm distilled water (40°C) onto microscope silane-coated slides (Super Frost Plus, Menzel-Glaser). Sections were allowed to dry overnight at 40°C. Prior to staining, tissue sections were deparaffinized in xylene and rehydrated by stepwise washes in decreasing ethanol/H_2_O ratio (96%, 70%) for 5 min in each and in distilled water. The slides were stained with hematoxylin solution (Mayer’s solution) for 10 min, washed in running water and stained with eosin. The slides were then treated with 70% ethanol for 20 s, 90% ethanol for 20 s, 100% ethanol for 1 min and xylene for 3 min dried, and mounted with xylene-based mounting media and coverslips (50).

### CVB3 virus titration of the organs suspension

To determine the replicating CVB3 titers in organs of infected mice Hep-2 cells were used. Cells were grown as monolayers in 96-well flat bottom plates in Minimum Essential Medium modified with Earle's Salts (MEM-E) supplemented with 1% HEPES, antibiotics and 10% FBS.

Serial 10-fold dilutions of the organ suspensions or of the serum samples with MEM-E (supplemented with 1% HEPES, antibiotics and 2% FBS) were made in 96-well U bottom plates (eight wells for each dilution) and 100 μl of diluted suspensions were transferred to the grown monolayers (the medium from the grown monolayers was first discarded). The plates were incubated in a CO_2_ incubator at 37°C and checked on days 4–5 under the light microscope. Titers were expressed as 50% tissue culture infectious dose (TCID_50_) following TCID_50_ values, calculated according to the Karber’s method (51).

### Plaque assay

To determine TBEV titers, A549 cells were used following a modified version of a previously described protocol (52). Here, 10-fold dilutions of the infectious samples were placed in 24-well plates and incubated with A549 cell suspension (1.2 × 10^5^ cell per well) for 4 h at 37°C and 5% CO2. The samples were then covered with an overlay mixture (1.5% carboxymethyl cellulose in complete culture media). After 5 days, the plates were washed with PBS and stained with naphthalene black. Virus-produced plaques were counted, and the titers were expressed as PFUs/ml. The EMCV titer was determined in L929 cells following the plaque assay methodology on a 96-well plate (53).

### Transfection

For transfection, cells were plated on a 24-well plate, grown to 50% density and transfected using Lipofectamine 3000 (Thermo Fisher Scientific) according to the manufacturer’s protocol.

### Western blot

Mouse organs and ES cells were homogenized mechanically in RIPA lysis buffer supplemented with 2× protease inhibitor cocktail set (Millipore) and loaded with sodium dodecyl sulfate dye. The protein concentration was measured by the Bradford assay (Bio-Rad) and 80 μg of total protein was used per lane. Proteins were separated in 5.5% polyacrylamide (PAA) gel and transferred onto polyvinylidene difluoride (PVDF) membrane (Millipore) using semi-dry blotting for 60 min, 35 V. The membrane was blocked in 5% skim milk in TBS-T, Dicer was detected using rabbit polyclonal anti-Dicer antibody #349 (54) (a gift from Witold Filipowicz, dilution 1:5000), anti-HA rabbit primary antibody (Cell Signaling, #3724, dilution 1:1000) and incubated overnight at 4°C. Secondary anti-Rabbit-HRP antibody (Santa Cruz Biotechnology, #sc-2357, dilution 1:50 000) was incubated for 1 h at room temperature. For TUBA4A detection, samples were separated in 10% PAA gel and incubated overnight at 4°C with anti-Tubulin (Sigma–Aldrich, #T6074, dilution 1:10 000). HRP-conjugated anti-mouse IgG binding protein (Santa Cruz Biotechnology, #sc-525409, dilution 1:50 000) was used for the detection. The signal was developed on films (X-ray Film Blue, Cole-Parmer, #21700-03) using the SuperSignal West Femto Chemiluminescent Substrate (Thermo Fisher Scientific).

### RNA isolation

Infected cells, uninfected cells and mouse organs were washed with PBS, homogenized in Qiazol lysis reagent (Qiagen), and total RNA was isolated by the Qiazol–chloroform extraction and ethanol precipitation method (55).

### RT-qPCR analysis

For Dicer expression analysis, aliquots of 3 μg of total RNA were treated by a TURBO DNA-free™ Kit (Invitrogen) according to the manufacturer’s instructions. Next, aliquots of 1 μg of total DNAse-treated RNA were used for complementary DNA (cDNA) synthesis by a LunaScript RT SuperMix Kit (New England Biolabs) according to the manufacturer’s instructions. For LCMV and EMCV RNA quantification, cDNA synthesis was performed directly after RNA isolation. A 1-μl cDNA aliquot and the Maxima SYBR Green qPCR Master Mix (Thermo Fisher Scientific) were used for the quantitative polymerase chain reaction (qPCR) reaction. qPCR was performed in LightCycler 480 (Roche) in technical triplicates for each biological sample. Average Ct values of the technical replicates were normalized using the ΔΔCt method to three housekeeping genes, *Hprt*, *Alas* and *B2mg* for mouse organs and *Hprt* or *B2mg* for mouse ESCs. *eEF1a1* was used as a housekeeping gene for one 3-dpi analysis of an LCMV infection of *Dicer^Δ HEL1/wt^*. A list of the primers used for qPCR is provided in the Supplementary Table S1.

### Isolation of RISC-associated small RNAs

ESCs infected with LCMV at a MOI of 0.1 or transfected with a plasmid expressing a MosIR dsRNA hairpin were used for isolation of RISC complexes by Trans-kingdom, rapid, affordable Purification of RISCs (TraPR; Lexogen, Austria) according to manufacturer’s instructions 24 and 48 hpi, respectively. Spleens infected with 2.5 × 10^6^ PFUs of LCMV Arm and collected at 3 dpi were also utilized for RISC purification by TraPR. RISC-associated small RNAs were extracted by a mixture of acidic phenol, chloroform and isoamyl alcohol and precipitated by cold isopropanol. RISC-associated small RNAs were immediately used for small RNA library preparation.

### Small RNA sequencing

After isolation, RNA quality was verified by electrophoresis in 1% agarose gel and RNA concentration was determined by the Qubit Broad Range Assay (Invitrogen). Small RNA libraries were prepared using a NEXTFLEX® Small RNA-Seq Kit v3 for Illumina (PerkinElmer) according to the manufacturer’s protocol; 3′ adapter ligation was performed overnight at 20 °C, 15 cycles were used for PCR amplification and gel purification was performed for size selection. For gel purification, libraries were separated in 2.5% agarose gel using 1× lithium borate buffer and visualized with ethidium bromide. The 150–160-bp fraction was cut off the gel and DNA was isolated using the MinElute Gel Extraction Kit (Qiagen). Final libraries were analyzed by an Agilent 2100 Bioanalyzer and sequenced by 75-nt single-end reading using the Illumina NextSeq500/550 platform.

### Bioinformatic analyses

RNA sequencing (RNA-seq) data (Supplementary Table S2) were deposited in the Gene Expression Omnibus database under accession number GSE273338.

#### Mapping of small RNA-seq data

Small RNA-seq reads were trimmed in two rounds using fastx-toolkit version 0.0.14 (https://github.com/agordon/fastx_toolkit) and cutadapt version 1.8.3 (56). First, four random bases were trimmed from the left side:

fastx_trimmer -f 5 -i {INP}.fastq -o {TMP}.fastq

Next, NEXTflex adapters were trimmed. Additionally, the *N* nucleotides on the ends of the reads were trimmed and reads containing >10% of the *N* nucleotides were discarded:

cutadapt –format=‘fastq’ –front=’GTTCAGAGTTCTACAGTCCGACGATCNNNN’ –adapter=’NNNNTGGAATTCTCGGGTGCCAAGG’ –error-rate = 0.075 –times = 2 –overlap = 14 –minimum-length = 12 –max-n = 0.1 –output=’${TRIMMED}.fastq" –trim-n –match-read-wildcards ${TMP}.fastq

The trimmed reads were mapped to the mouse (mm10) genome with the following parameters:

STAR –readFilesIn ${TRIMMED}.fastq.gz –runThreadN 4 –genomeDir ${GENOME_INDEX} –genomeLoad LoadAndRemove –readFilesCommand unpigz -c –readStrand Unstranded –limitBAMsortRAM 20000000000 –outFileNamePrefix ${FILENAME} –outReadsUnmapped Fastx –outSAMtype BAM SortedByCoordinate –outFilterMultimapNmax 99 999 –outFilterMismatchNoverLmax 0.1 –outFilterMatchNminOverLread 0.66 –alignSJoverhangMin 999 –alignSJDBoverhangMin 999

#### Viral small RNA analyses

Small RNA-seq reads were trimmed in two rounds using bbduk.sh version 38.87 (57). First, NEXTflex adapters were trimmed from the right end:

bbduk.sh -Xmx20G threads = 6 in=${FILE}.fastq.gz out=${FILE}.atrim.fastq.gz literal = TGGAATTCTCGGGTGCCAAGG stats=${FILE}.atrim.stats overwrite = t ktrim = r k = 21 rcomp = f mink = 10 hdist = 1 minoverlap = 8

Next, four random bases from both sides of reads were trimmed:

bbduk.sh -Xmx20G threads = 6 in=${FILE}.atrim.fastq.gz out=${FILE}. trimmed.fastq.gz stats=${FILE}.ftrim.stats overwrite = t forcetrimright2 = 4 forcetrimleft = 4 minlength = 18

The genome index was created by joining the mouse genome .fasta file (GCA_000001635.2, mm10) with individual viral genomes (JN048469.1, NC_001479.1, NC_004291.1, NC_004294.1 and U39292.1). The trimmed reads were mapped to such genome index with STAR version 2.7.10b (58) with the following parameters:

STAR –readFilesIn ${FILE}.fastq.gz –genomeDir ${GENOME_INDEX} –runThreadN 12 –genomeLoad LoadAndRemove –limitBAMsortRAM 20000000000 –readFilesCommand unpigz –c –outFileNamePrefix ${FILENAME} –outSAMtype BAM SortedByCoordinate –outReadsUnmapped Fastx –outFilterMismatchNoverLmax 0.1 –outFilterMatchNmin 16 –outFilterMatchNminOverLread 0 –outFilterScoreMinOverLread 0 –outFilterMultimapNmax 99 999 –outFilterMultimapScoreRange 0 –alignIntronMax 1 –alignSJDBoverhangMin 999 999 999 999

For viral genome coverage, only reads of length 21–23 nt were visualized. If reads were aligned to the viral genomes with ≤3 soft-clipped nucleotides alignment, such soft-clipped nucleotides were added to the read lengths.

The mapped reads were counted using program featureCounts (59). Only reads with length 18–32 nt were selected from the small RNA-seq data:

featureCounts -a ${ANNOTATION_FILE} -F ${FILE} -minOverlap 15 -fracOverlap 0.00 -s 1 -M -O -fraction -T 8${FILE}.bam

The GENCODE gene set (60) was used for the annotation of long RNA-seq data. The miRBase 22.1. (61) set of miRNAs was used for the annotation of small RNA-seq data. Statistical significance and fold changes in gene expression were computed in R using the DESeq2 package (62). Genes were considered to be significantly up- or down-regulated if their corresponding *P*-adjusted values were <0.05.

### Statistical analyses

For statistical testing, a two-tailed *t*-test was used.

## Results

### Absence of antiviral effects in *Dicer^Δ HEL1/wt^* mice

We set out to investigate whether a physiologically expressed Dicer^ΔHEL1^ variant supports innate antiviral immunity. To this end, we exposed *Dicer^Δ HEL1/wt^* mice to four different viruses (picornaviruses CVB3 and EMCV, flavivirus TBEV and arenavirus LCMV) and investigated whether these viruses were recognized and targeted by enhanced RNAi in *Dicer^Δ HEL1/wt^* mice.

### CVB3 (strain Nancy)

The first tested virus was the CVB3 [strain Nancy (63)], a (+)ssRNA virus that belongs to the genus *Enterovirus* of the family *Picornaviridae* (64). Infections of *Dicer^Δ HEL1/wt^* mice (CD1 outbred background) were performed in collaboration with the Enterovirus Laboratory at the Slovak Medical University. In the first experiment, 4-week-old mice (five mice per genotype and timepoint) were infected with 0.5 ml of 10^5^ TCID_50_/ml by intraperitoneal injection and organs were collected at 3 and 5 dpi. We observed significant reduction of the viral titer in *Dicer^Δ HEL1/wt^* animals (Figure 1B). This finding was corroborated by histology and RT-qPCR (reverse transcription quantitative real-time PCR) analysis of viral RNA in the infected mice, where mild to severe infiltration was observed in the acinar cells of the pancreatic tissue as compared with the uninfected controls where the infiltration was absent (Supplementary Figure S1A). However, this reduction in viral copies and replicating viruses was not reproduced in any subsequent infection of 4-week-old (Figure 1C) and 3-week-old mice (Figure 1D). Furthermore, we did not detect virus-derived 21–23-nt siRNAs in the infected spleens (viral titer ∼10^4^ plaques/g) of juvenile mice. Putative vsiRNAs were not detectable at the sequencing depth of 20 million reads (Figure 1E and F, and Supplementary Figure S1B).

### EMCV

**Figure 2.**
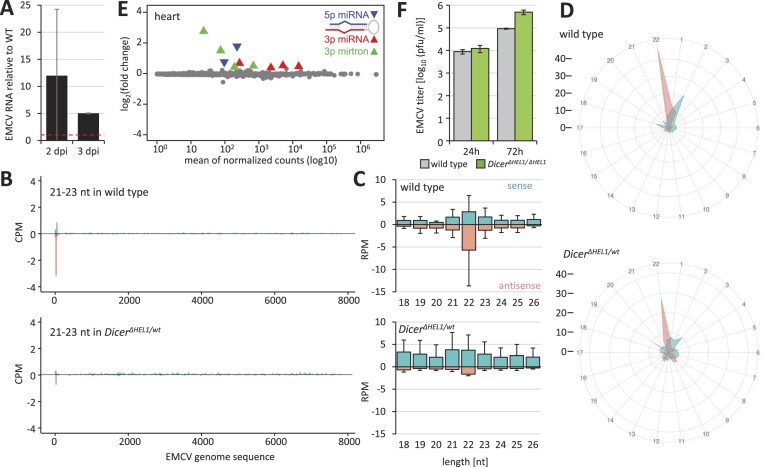
Infection of *Dicer^Δ HEL1/wt^* with EMCV (*Picornaviridae*). (**A**) EMCV viral RNA in the hearts of infected *Dicer^Δ HEL1/wt^* 6-week-old mice is several folds higher than in wild type littermates. Animals were infected subcutaneously with 10^3^ PFUs (2 dpi: three animals per genotype; 3 dpi: two animals per genotype). Error bars = SD for 2 dpi, range of two values for 3 dpi. (**B**) A coverage plot for EMCV 21–23-nt small RNAs represents combined data for two different infected hearts 3 dpi for sense (+) and antisense (−) small RNAs mapped to the viral genome sequence. (**C**) Distribution of frequencies of small RNAs of different lengths mapping onto the EMCV genome in the infected hearts (*n* = 2, 3 dpi). Error bars = range of values. (**D**) Phasing analysis of 21–23-nt RNAs mapping onto the EMCV genome sequence. The viral genome sequence was divided into 22 possible phased registers in both, sense and antisense orientation, and 21–23-nt reads mapping to the sequence were counted in these registers based on the position of their 5′ nucleotide. Abundance of reads in each register is displayed as the distance from the center, which indicates read percentage within each register. The first 5′ EMCV nucleotide defines the register no. 1. Radar plots show 21–23-nt reads assigned to 22 possible registers along the entire EMCV sense (iris blue) and antisense (salmon) strands in wild type and *Dicer^Δ HEL1/wt^* infected hearts. (**E**) MA plot of differentially expressed miRNAs in *Dicer^Δ HEL1/wt^* EMCV-infected hearts relative to wild type infected hearts; for each genotype *n* = 3. (**F**) A pilot experiment of EMCV viral titers (PFU/ml) in wild type and *Dicer^Δ HEL1/^*^ΔHEL1^ ESCs 24 and 72 hpi. The experiment was performed in triplicate. Error bars = SD.

The second tested virus was the picornavirus EMCV, which has the tropism for the heart [reviewed in (65)] and whose targeting by RNAi was reported in ESCs (26). EMCV was a strong candidate for testing antiviral RNAi because efficient RNAi and endo-siRNA production was observed in the hearts of *Dicer^Δ HEL1/wt^* mice (43). In collaboration with the Laboratory of Arbovirology from the Biology Centre of the Czech Academy of Sciences, we performed a pilot experiment with three C57Bl/6 young adult mice per group injected subcutaneously with 10^3^ PFUs and we tested progression of the EMCV infection at 2 and 3 dpi. However, analysis of RNA from the infected hearts showed several fold increased levels of viral RNA in *Dicer^Δ HEL1/wt^* mice relative to the wild type siblings (Figure 2A). Small RNA-seq of infected hearts at 3 dpi showed low amounts of 21–23-nt reads mostly localized to the 5′ end of the viral genome sequence (Figure 2B), reminiscent of siRNA production from blunt-end substrates in mammalian cells (13) and consistent with EMCV siRNA distribution along the viral sequence from infected ESCs (26). Processing of EMCV into vsiRNAs was supported by a minor peak of 22-nt RNA species in RNA-seq data in wild type and *Dicer^Δ HEL1/wt^* mice (Figure 2C) and phasing analysis showing a positive signal for sense 21–23-nt RNA in the 22-nt register and a 2-nt shift for antisense 21–23-nt RNA, which corresponds to the published results from infected ESCs (26) (Figure 2D). However, the abundance of putative EMCV vsiRNAs was low, which contrasted with a three orders of magnitude higher abundance of vsiRNAs observed in the infected ESCs (26). Furthermore, there was no increased abundance of EMCV-derived 21–23-nt RNAs in *Dicer^Δ HEL1/wt^* mice relative to the wild type mice (Figure 2C). This contrasted with efficient siRNA production from expressed dsRNA in the *Dicer^Δ HEL1/wt^* mice (43) and suggested poor accessibility/processing of the viral dsRNA by Dicer *in vivo*. Unexpectedly, the increased Dicer activity *in vivo* in the heart apparently promoted the viral infection as indicated by the increased amount of EMCV RNA in the heart (Figure 2A). The cause of the pro-viral effect is unclear but appears miRNA-independent, as there were minimal miRNome changes in *Dicer^Δ HEL1/wt^* hearts of the infected animals (Figure 2E), which concerned primarily low-expressed mirtrons and miRNA passenger strands. Because of the pro-viral effect, we did not investigate the EMCV *in vivo* infection model further. This decision was confirmed by a pilot experiment in *Dicer^Δ HEL1/ Δ HEL1^* ESCs (6), which also failed to support increased antiviral RNAi (Figure 2F).

### TBEV

**Figure 3.**
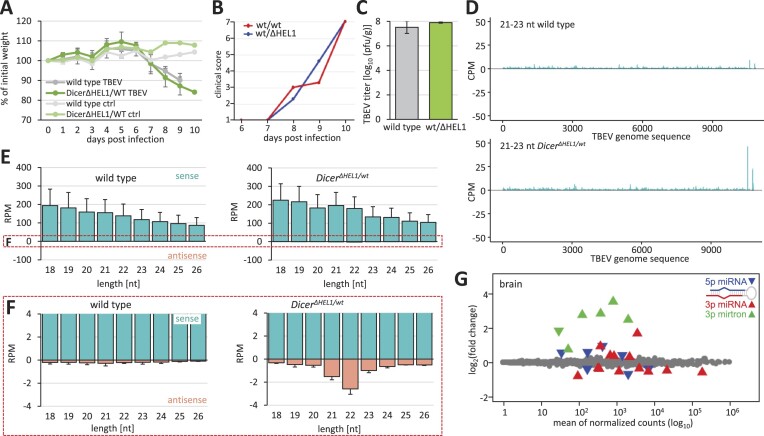
Infection of *Dicer^Δ HEL1/wt^* and wild type mice with TBEV (*Flaviviridae*). Animals were infected subcutaneously with 10^3^ PFU. (**A**) Loss of weight of infected animals. *n* = 3. Error bars = SEM. (**B**) Development of severity of infection. Clinical score 1 = healthy, 2 = piloerection, 3 = hunched back, 4 = paralysis (one leg), 5 = paralysis (two legs), 6 = moribund and 7 = dead. (**C**) TBEV viral titers obtained from the infected brains of animals of the specified genotypes at 10 dpi (7-week old, *n* = 3 animals per genotype/timepoint). Error bars = SD. (**D**) Coverage plots for TBEV 21–23-nt small RNAs in the brains of *Dicer^Δ HEL1/wt^* and wild type mice at 10 dpi. Both panels combine data from three different infected brains. (**E**) Distribution of frequencies of small RNAs of different lengths mapping onto the TBEV genome in the infected brains (*n* = 3; 10 dpi). Error bars = SD. (**F**) The same data as in panel (E) but shown at a scale where 21–23-nt antisense RNAs are visible. Antisense 21–23-nt RNAs in *Dicer^Δ HEL1/wt^* are significantly more abundant than in wild type brains (two-tailed *t*-test *P*-value < 0.05). (**G**) MA plot of differentially expressed miRNAs in *Dicer^Δ HEL1/wt^* TBEV infected brains relative to wild type infected brains; for each genotype, *n* = 3.

The third tested virus was TBEV. In collaboration with the Laboratory of Arbovirology, we conducted two experiments with young adult C57Bl/6 mice (6–7-week old) infected by subcutaneous injection of TBEV (10^3^ PFUs). *Dicer^Δ HEL1/wt^* mice lost weight and developed symptoms comparably to wild type animals (Figure 3A and B). There was no difference in the TBEV titer between *Dicer^Δ HEL1/wt^* and wild type controls at 10 dpi (Figure 3C). It was reported that TBEV and related flaviviruses produced virus-derived 22-nt small RNAs in tick cells (45), but RNA-seq analysis of the mouse brain at 10 dpi provided little evidence for TBEV vsiRNAs. TBEV-derived 21–23-nt RNAs were mostly coming from the sense strand and we observed their increased abundance in a specific 3′ end region of the viral genomic sequence in *Dicer^Δ HEL1/wt^* brains (Figure 3D). However, the size distribution of RNA fragments did not show enrichment of 21–23-nt RNAs in comparison with longer and shorter small RNAs in wild type or *Dicer^Δ HEL1/wt^*-infected brains (Figure 3E), suggesting that small RNAs from the sense strand are mostly degradation fragments. Notably, analysis of antisense small RNAs, which were two orders of magnitude less abundant than the sense fragments, identified a distinct peak of 21–23-nt RNAs derived from the minus strand in *Dicer^Δ HEL1/wt^* brains (Figure 3F). This peak was not observed in TBEV-infected wild type brains (Figure 3F). This implied that the RNAi machinery in the brain of *Dicer^Δ HEL1/wt^* mice cleaves TBEV dsRNA into low-abundant siRNAs, which do not significantly affect viral replication.

Analysis of miRNA expression in *Dicer^Δ HEL1/wt^* mutants showed highly selective miRNA changes, which did not support general suppression of the Dicer function (Figure 3G). In fact, increased mirtron levels in the brains of infected *Dicer^Δ HEL1/wt^* mice but not in wild type animals document increased activity of Dicer^ΔHEL1^ (Figure 3G).

**Figure 4.**
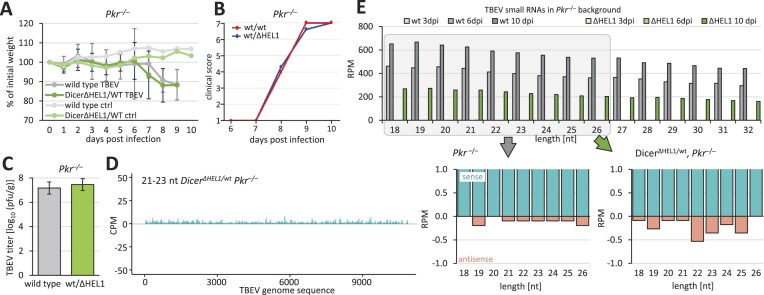
Infection of *Dicer^Δ HEL1/wt^ Pkr^–/–^*and wild type mice with TBEV (*Flaviviridae*). Animals were infected subcutaneously with 10^3^ PFU. (**A**) Loss of weight of infected animals. *n* = 3. Error bars = SEM. (**B**) Development of severity of infection. Clinical score 1 = healthy, 2 = piloerection, 3 = hunched back, 4 = paralysis (one leg), 5 = paralysis (two legs), 6 = moribund and 7 = dead. (**C**) TBEV viral titers obtained from the infected brains of animals of the specified genotypes at 10 dpi (7-week old, *n* = 3 animals per genotype/timepoint). Error bars = SD. (**D**) Coverage plots for TBEV 21–23-nt small RNAs in the brains of *Dicer^Δ HEL1/wt^ Pkr^–/–^* mice at 10 dpi. The panel shows RNA-seq data from a single infected brain. (**E**) The abundance of reads of different lengths in infected wild type and *Dicer^Δ HEL1/wt^* brains lacking functional PKR. The lower graphs depict 18–26-nt distribution of sense and antisense RNAs in wild type and *Dicer^Δ HEL1/wt^* brains lacking functional PKR at 10 dpi.

Finally, we also tested whether the absence of protein kinase R (PKR, official symbol EIF2AK2), a key innate immunity dsRNA sensor, would impact the virus targeting by RNAi *in vivo*. The rationale for this experiment stemmed from the fact that experiments in cell culture (HEK293T, 3T3 and ES cells) reported that the loss of PKR stimulated siRNA production and RNAi (13,43,66). We infected wild type and *Dicer^Δ HEL1/wt^* mice, which were also homozygous for deletion of dsRNA binding-domain encoding exons of *Pkr*. *Pkr* mutants showed a similar loss of weight as mice with normal *Pkr* (Figure 4A versus 3A), but died one day earlier (Figure 4B). *Pkr* mutants reached the same TBEV viral titers in the post-mortem brain as their *Pkr* wild type counterparts (Figure 4C versus 3C) and there was no significant difference in the viral titer in the post-mortem brain between wild type and *Dicer^Δ HEL1/wt^* in the *Pkr* mutant background (Figure 4C). Small RNA-seq of 3, 6 and 9 dpi samples did not support TBEV targeting by RNAi, as 21–23-nt TBEV-derived RNAs had the aforementioned strong asymmetry toward the (+) strand and did not show clear enrichment of 21–23-nt small RNA species (Figure 4E).

### LCMV

**Figure 5.**
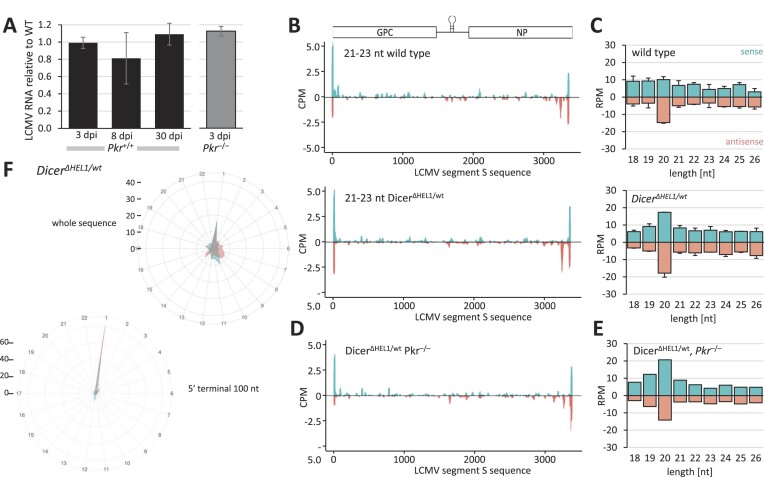
Infection of *Dicer^Δ HEL1/wt^* with LCMV (*Arenaviridae*). (**A**) LCMV RNA in the spleens from 11–17-week-old *Dicer^Δ HEL1/wt^* and *Dicer^Δ HEL1/wt^ Pkr*^-/-^ animals infected with LCMV at 3, 8 or 30 dpi relative to wild type (*n* = 3 animals per genotype/timepoint). Animals were injected with 2 × 10^5^ PFUs LCMV Arm except for the 30 dpi timepoint where animals were injected with 10^6^ PFUs of LCMV C13 intravenously. Error bars = SEM. (**B**) Coverage plots for 21–23-nt small RNAs mapping to the S segment of the LCMV genome. Each plot depicts combined data obtained from two different spleens at 3 dpi. (**C**) Analysis of the length distribution of small RNAs derived from the segment S at 3 dpi in the spleens of infected *Dicer^Δ HEL1/wt^* and wild type animals (*n* = 2; error bars = range of values). (**D**) Analysis of small RNAs from the infected spleen of a *Dicer^Δ HEL1/wt^ Pkr*^-/-^ mouse, which was infected with 2 × 10^5^ PFU of LCMV Arm and collected at 3 dpi. Shown is a coverage plot for 21–23-nt small RNAs mapping to the S segment. (**E**) Analysis of the length distribution of sense and antisense small RNAs derived from viral RNA in the spleens of infected *Dicer^Δ HEL1/wt^ Pkr*^-/-^ mice at 3 dpi. (**F**) Phasing analysis of 21–23-nt RNAs mapping onto the LCMV S segment. The upper right diagram is based on analysis of all mapped 21–23-nt RNAs, while the lower left one took into account only those mapping to 100 nt at the 5′ terminus of the genomic RNA.

The final *in vivo* tested virus was LCMV, a segmented (−)ssRNA virus from the family *Arenaviridae* with the tropism for secondary lymphoid organs [reviewed in (67)]. This work was done in collaboration with the Laboratory of Adaptive Immunity from the Institute of Molecular Genetics of the Czech Academy of Sciences. The virus was delivered by intraperitoneal injection and its levels in the spleen were not significantly reduced in either *Dicer^Δ HEL1/wt^* or *Dicer^Δ HEL1/wt^ Pkr^–/–^* mutants (Figure 5A).

Similarly to the EMCV infection, we observed 21–23-nt small RNAs generated from sense and antisense strands, particularly at the termini of viral genomic RNAs (Figure 5B, and Supplementary Figure S2). However, small RNA analysis showed no specific enrichment of 21–23-nt small RNAs among 18–32-nt small RNAs and there was a similar abundance of putative vsiRNAs in wild type animals and *Dicer^Δ HEL1/wt^* mutants (Figure 5C). Loss of PKR did not have any positive effect on enrichment of 21–23-nt small RNAs (Figure 5D and E).

While the size distribution of LCMV-derived small RNAs in the spleen had a distinct 20-nt, but not a 22-nt, peak (Figure 5C and E), phasing analysis of 21–23-nt small RNAs showed a weak signal in the register 1 for both, sense and antisense 21–23-nt RNAs in the entire sequence (Figure 5F). This signal became much stronger when the analysis was restricted only to the terminal 100 nt (Figure 5F) suggesting that some fractions of 21–23-nt reads from the termini might be produced by Dicer.

**Figure 6.**
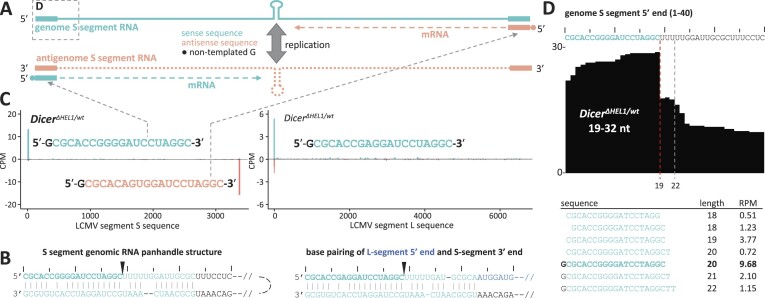
Most abundant small RNAs from LCMV are 20-nt long and originate from 5′ ends of viral mRNAs. (**A**) A scheme of the relationship of different LCMV RNAs from the S segment. The genome (−ssRNA) and sequences in the same orientation are depicted in the iris blue color, antisense sequences to them, such as the antigenome and mRNAs, are depicted in the salmon color. The genome and antigenome are transcribed from the 5′ end into mRNAs, which carry a non-templated G addition depicted as a circle. The rectangles at the termini symbolize complementary terminal sequences. (**B**) Predicted base pairing between terminal sequences of the genomic RNA of the S segment and base pairing between the 3′ end of the S segment and the 5′ end of the L segment. The bold font and the black triangle indicate the 5′ terminal 19 nt of the S and L segments, which represent the most abundant mapped viral genomic sequence in the small RNA-seq samples. (**C**) Visualization of 19-nt-mapped RNA fragments identified the terminal sequences as the origin of these RNA but analysis of the real length of these RNAs identified that they are 20-nt long and carry a non-templated G addition at their 5′ end (depicted in black font) suggesting that these fragments originate from LCMV mRNAs. (**D**) A UCSC browser snapshot of the 5′ end of the S segment with mapped 18–32-nt RNA-seq data and most abundant small RNAs matching this region with RPM > 0.5 sorted by size. Clearly visible is the sharp ending after the genomic nucleotide 19th, which corresponds to the high abundance of 20-nt RNA species with the 5′ G addition. Putative siRNAs of 21–23-nt length are much less abundant than the 20-nt fragment. A similar situation occurs at the 3′ end of the S segment and the 5′ end of the L segment. The top ten most abundant 18–32 RNAs from the S segment sequenced from the spleens and ESCs of different genotypes are presented in Supplementary Figure S3.

To understand the origin of the 20-nt peak in the small RNA size distribution, we reviewed the origin of the most abundant LCMV-derived small RNAs. LCMV has a complex RNA cycle during which dsRNA may arise (Figure 6A). Replication of small (S) and large (L) segments produces complementary genome and antigenome RNAs, which are transcribed from their 3′ ends into messenger RNAs (mRNAs) carrying non-templated 5′ nucleotide additions (68). In addition, internal base pairing can occur at the ends of the S segment RNAs, as the terminal ∼30 nt of the S segment are highly complementary and can form an intramolecular dimer. In addition, the 3′ end of the S segment genome is complementary to the 5′ end of the L segment and can form a duplex as well (Figure 6B).

Analysis of the most abundant small RNA reads revealed that 20-nt fragments responsible for the increased frequency of this length in the spleen samples are derived from 5′ ends of viral mRNAs as they carry a non-templated G addition at the 5′ end followed by 19-nt mapping to the viral genomic sequence (Figure 6C, and Supplementary Figure S3). In RNA-seq data mapped to the genome, these reads formed a distinct boundary at the 19th nucleotide position (Figure 6D) and their abundance was much higher than that of putative 21–23-nt Dicer products (Figure 6D, and Supplementary Figure S3). We hypothesize that a large fraction of these 20-nt RNA fragments may be degradation products of viral mRNAs rather than Dicer products because of their shorter length and presence in *Dicer^–/–^* ESCs (Supplementary Figure S3).

### Higher levels of truncated Dicer variants target LCMV in ESCs

**Figure 7.**
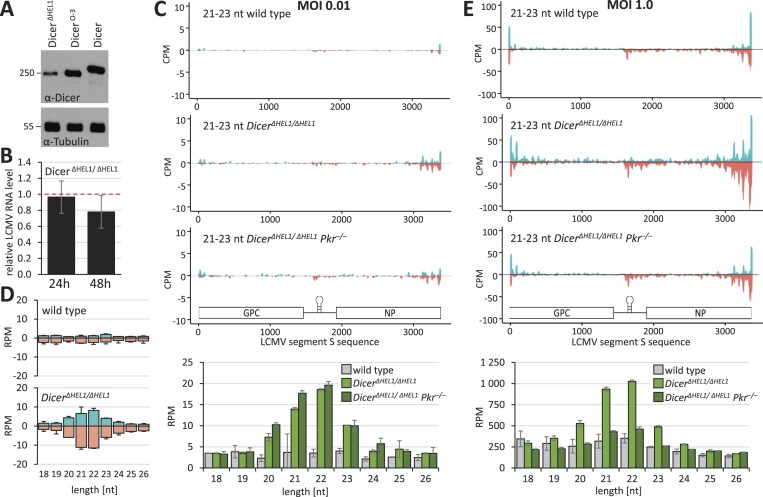
Analysis of ESC lines expressing truncated Dicer. (**A**) Western blot analysis of Dicer protein levels in a Dicer^ΔHEL1^ homozygote, Dicer^O-3^ ESC line and unmodified ESCs (Dicer). (**B**) LCMV RNA levels 24 and 48 hpi (MOI = 0.01) in *Dicer^Δ HEL1/ Δ HEL1^* ESCs relative to the infected parental ESC line. Error bars = SEM. (**C**) Coverage plots show 21–23-nt reads from the infected ESCs (MOI = 0.01) mapped onto the S segment of the LCMV genome. From above: the parental ESCs line (*Dicer^wt/wt^*), *Dicer^Δ HEL1/wt^* and *Dicer^Δ HEL1/wt^ Pkr*^-/-^ [the same cell lines were studied in (43), Figure 3]. The analysis was performed in a duplicate infection; coverage plots display combined data. The histogram below the coverage plots shows analysis of the size distribution of small RNAs. Error bars = range of values. (**D**) Analysis of the length distribution of sense and antisense small RNAs derived from viral RNA in wild type and *Dicer^Δ HEL1/ Δ HEL1^* ESCs (*n* = 2; 24 hpi). Error bars = range of values. (**E**) Coverage plots of 21–23-nt reads from ESCs infected at MOI 1.0.

Because RNAi in *Dicer^Δ HEL1/wt^* was insufficient to inhibit LCMV *in vivo*, we turned to ESCs to investigate how the Dicer activity may support/limit the antiviral effects. We employed two ESC lines with high Dicer activity produced in the lab previously: *Dicer^Δ HEL1/ Δ HEL1^* (6,43) and *Dicer^O-3^* ESC lines (17) (Figure 7A).

Infection of the *Dicer^Δ HEL1/ Δ HEL1^* ESC lines at MOI 0.01 showed a minor (22.1%) but statistically significant reduction of LCMV RNA (Figure 7B). The general profile of virus-derived 21–23-nt small RNAs also showed a higher signal around the termini of the viral genome (Figure 7C). In contrast to normal ESCs, there was a clear enrichment of LCMV-derived 21–23-nt RNAs in *Dicer^Δ HEL1/ Δ HEL1^* ESCs (Figure 7C and D). We also examined siRNA production in *Dicer^Δ HEL1/ Δ HEL1^* ESCs in the absence of PKR and at a hundred times higher MOI (1.0). Similarly to experiments in mice with TBEV and LCMV (Figures 4 and 5A), the absence of PKR did not enhance vsiRNA production in ESCs (Figure 7C and E). At MOI 1.0, putative vsiRNAs mapped along the viral genome and reached ∼2500 RPM reads, which was ∼50× higher relative to MOI 0.01 (Figure 7E). This implied that the relatively low vsiRNA abundance observed at MOI 0.01 was mainly a consequence of low substrate availability.

**Figure 8.**
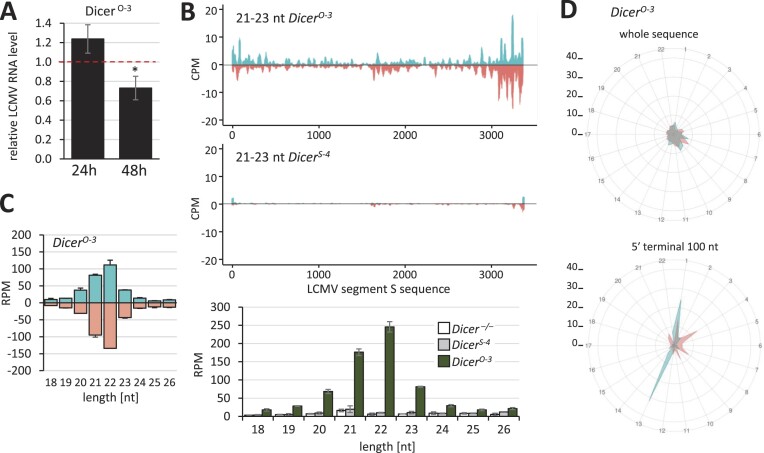
Antiviral effects in an ESC line expressing high levels of truncated Dicer. *Dicer^S-4^* and *Dicer^O-3^* are stable ESC lines where the Dicer expression in *Dicer^–^*^/^*^–^* (69) was rescued with stable expression of the full-length Dicer (S4) and shorter Dicer^O^ variant (O3), respectively. Both Dicer variants have a comparable level of expression (17). (**A**) LCMV RNA levels 24 and 48 hpi (MOI = 0.01) in the Dicer^O-3^ ESC line relative to Dicer^S-4^. Error bars = SEM. * *P*-value < 0.05. (**B**) 21–23-nt LCMV abundance in *Dicer^O-3^* and *Dicer^S-4^* ESCs. The coverage plot for 21–23-nt small RNAs mapping to the S segment of the LCMV genome depicts data from infected Dicer^O-3^ ESCs at 24 hpi (MOI = 0.01). The analysis was performed in a duplicate infection, the coverage plots display combined data. The histogram below the coverage plots shows analysis of the size distribution of small RNAs and includes parental *Dicer^–^*^/^*^–^* analysis. (**C**) Analysis of the length distribution of sense and antisense small RNAs derived from viral RNA in *Dicer^O-3^* ESCs (*n* = 2; 24 hpi). Error bars = range of values. (**D**) Phasing analysis of 21–23-nt RNAs from infected *Dicer^O-3^* ESCs mapping onto the LCMV sequence. The upper diagram is based on analysis of all mapped 21–23-nt RNAs, while the lower one took into account only those mapping to 100 nt at the 5′ terminus of the genomic RNA.

To further examine how Dicer expression levels influence vsiRNA production, we employed the *Dicer^O-3^* ESC line. It carries out a stable expression of the oocyte-specific Dicer^O^ variant in the absence of expression of endogenous Dicer (17) and expresses even more truncated Dicer than *Dicer^Δ HEL1/ Δ HEL1^* ESCs (Figure 7A). Infection of the *Dicer^O-3^* line at MOI 0.01 showed a statistically significant reduction of LCMV RNA by 26.9% (Figure 8A) and an increased abundance of LCMV-derived 21–23-nt RNAs in *Dicer^O-3^* ESCs, which was ∼10× higher than in the *Dicer^Δ HEL1/ Δ HEL1^* ESCs (Figure 8B and C versus 7C and D). This suggests that there was still enough LCMV dsRNA available at MOI 0.01 for an order of magnitude higher vsiRNA production when the expression of a truncated Dicer variant ramped further up in the *Dicer^O-3^* line.

Phasing analysis of *Dicer^O-3^* ESCs showed a strong signal in the register 1 for both, sense and antisense 21–23-nt RNAs when the analysis was restricted to the terminal 100 nt (Figure 8D). Interestingly, a second signal on the sense strands came from the register 13 (Figure 8D). We hypothesize that this signal may come from a sense strand, which was cleaved by antisense siRNA but still replicated by the viral RNA polymerase, thus creating a blunt end dsRNA starting at this position. Notably, when the whole S segment sequence was used, phasing analysis of 21–23-nt RNAs from *Dicer^O-3^* ESCs did not show a dominant signal in any register (Figure 8D). Considering that RNA-seq from *Dicer^O-3^* ESCs showed 21–23-nt LCMV-derived small RNAs mapping along the S segments in both directions (Figure 8B), it seems that in addition to phased 21–23-nt RNAs produced from its termini, the abundant highly active Dicer also initiates LCMV dsRNA processing by stochastic endonucleolytic cleavage (5), thus generating a pool of vsiRNAs, which would not match a specific register.

**Figure 9.**
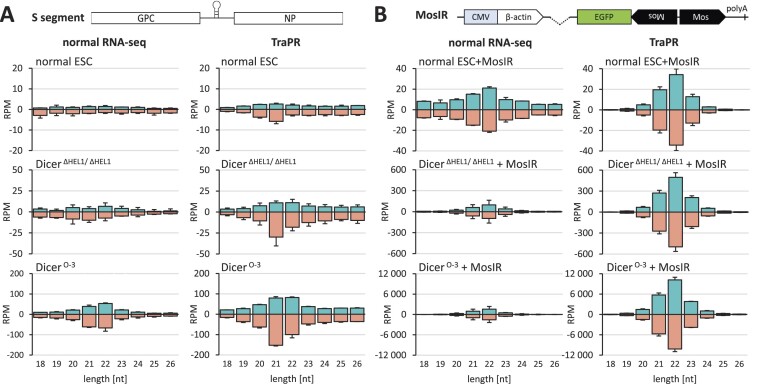
Analysis of Argonaute association with LCMV-derived small RNAs. (**A**) LCMV siRNAs. RNA-seq was performed in parallel from total RNA isolated from infected cells 24 hpi (MOI = 0.1, *n* = 2) either directly (left panel) or with the TraPR method to isolate AGO-associated small RNAs (right panel). (**B**) Long dsRNA-derived siRNAs. Small RNA length distribution from parental wild type, *Dicer^Δ HEL1/ Δ HEL1^* and *Dicer^O-3^* ESCs transfected with a plasmid encoding the MosIR dsRNA hairpin (24 h post transfection, *n* = 2). Samples were sequenced directly (left panel) or with the TraPR method (right panel). Error bars in panels = range of values.

To test whether putative LCMV-derived vsiRNAs are loaded onto RISC, we compared normal small RNA-seq with sequencing of small RNAs isolated with the TraPR isolation method (Figure 9), which purifies RNAs associated with the RISC effector complex (70). As a positive control for siRNA production, we included ESCs transfected with a MosIR plasmid, which expresses a long dsRNA hairpin activating RNAi (17,71). After sequencing, we observed a peak of 21–23-nt RNAs in the TraPR-isolations suggesting that these small RNAs were indeed bona fide LCMV-derived vsiRNAs. The enrichment of 21–23-nt RNAs in TraPR was ∼2-fold for LCMV-derived small RNAs (Figure 9A). Interestingly, 21 nt appeared the most common length of vsiRNAs isolated with TraPR (Figure 9A) while TraPR-isolated siRNAs from the long dsRNA hairpin peaked at 22 nt (Figure 9B). This difference is due to abundant 21-nt RNAs from the 5′ ends of LCMV mRNAs, which skew the vsiRNA distribution.

Consistent with higher expression of a truncated Dicer variant, *Dicer^O-3^* ESCs yielded about an order of magnitude more vsiRNAs with TraPR (Figure 9A). At the same time, MosIR 21–23-nt RNAs from TraPR isolation were more enriched and much more abundant in *Dicer^Δ HEL1/ Δ HEL1^* and *Dicer^O-3^* ESCs (Figure 9B). MosIR siRNAs were detectable in normal ESCs at an abundance similar to that of LCMV siRNAs in *Dicer^Δ HEL1/ Δ HEL1^* ESCs (Figure 9). In comparison with normal ESCs, *Dicer^Δ HEL1/ Δ HEL1^* and *Dicer^O-3^* ESCs exhibited one and two orders of magnitude higher MosIR siRNA levels, respectively. This result demonstrates the high capacity to produce siRNAs in *Dicer^Δ HEL1/ Δ HEL1^* and *Dicer^O-3^* ESCs. Taken together, ESC infections with LCMV showed that the increased abundance of a Dicer variant lacking the HEL1 domain induces vsiRNA production and repression of LCMV.

### Higher Dicer^ΔHEL1^ expression is antiviral *in vivo*

**Figure 10.**
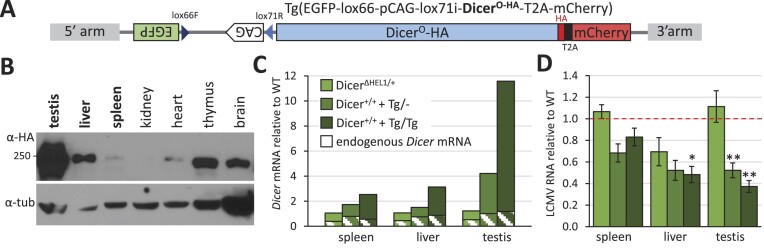
Antiviral effects in *Dicer^Tg(O-HA)^* transgenic mice overexpressing truncated Dicer. (**A**) A scheme of the inducible Dicer transgene. (**B**) Leaky transgene expression in *Dicer^Tg(O-HA)^* mice. Even though the CAG promoter is oriented antisense to the *Dicer* coding sequence, the transgene has leaky Dicer expression, which varies among organs. The expression differs from the CAG promoter expression pattern observed in other transgenes (43,73,74). The western blot was adopted from (44) for the reader’s convenience. (**C**) Estimation of total and endogenous *Dicer* mRNA levels in the spleen, liver and testis of mice with the three indicated genotypes. RNA from organs of one animal was analyzed by RT-qPCR in a technical triplicate, shown is the median value. The white-hatched parts in the bars correspond to the estimated level of the endogenous full-length Dicer mRNA amplified by a different set of primers. (**D**) LCMV RNA in the indicated organs from 11–12-week-old infected *Dicer^Δ HEL1/wt^*, *Dicer^+/+^+ ^Tg/-^* and *Dicer^+/+^+ ^Tg/Tg^* animals relative to wild type littermates. Data are from two independent experiments; three animals per genotype per experiment were injected with 2.5 × 10^6^ PFU of LCMV Arm intraperitoneally, organs were collected at 3 dpi. Error bars = SEM. * and ** indicate the *P*-values <0.05 and <0.01 from a two-tailed *t*-test, respectively.

To test whether LCMV could be affected *in vivo* by increasing the Dicer activity beyond that achieved in *Dicer^Δ HEL1/wt^*, we took advantage of the transgene Tg(EGFP-lox66-pCAG-lox71i-Dicer^O-HA^-T2A-mCherry), for simplicity referred to as *Dicer^Tg(O-HA)^* hereafter (Figure 10A). This transgene was produced for Cre-inducible expression of C-terminally HA-tagged Dicer^O^ (Dicer^O-HA^) from the CAG promoter (44). However, analysis of the transgenic mouse line revealed that the uninduced allele had leaky expression, which varied across organs (Figure 10B) and differed from the previously reported expression of CAG (72,73). The leakage was the highest in the testes, where it was observed in meiotic and post-meiotic cells (44). Analysis of total Dicer mRNA expression in different organs of animals carrying one or two copies of the transgene suggested a one–three times increased *Dicer* transcript level, except for the testis, where the leakage was much stronger (Figure 10C). These data suggested that *Dicer^Tg(O-HA)^* mice homozygous for the transgene express a higher truncated Dicer level than *Dicer^Δ HEL1/wt^* mice, and we thus examined how they would respond to LCMV infection.

Remarkably, when we infected mice carrying one or two copies of the *Dicer^Tg(O-HA)^* transgene, we observed 20–30% reduction of LCMV RNA levels in the spleen and 50–60% reduction in the liver and testes (Figure 10D). The difference was statistically significant in transgenic homozygotes in the liver and testes and in transgenic hemizygotes in the testes (Figure 10D). This is a relatively weak antiviral effect considering that strong antiviral effects reduce viruses by orders of magnitude.

**Figure 11.**
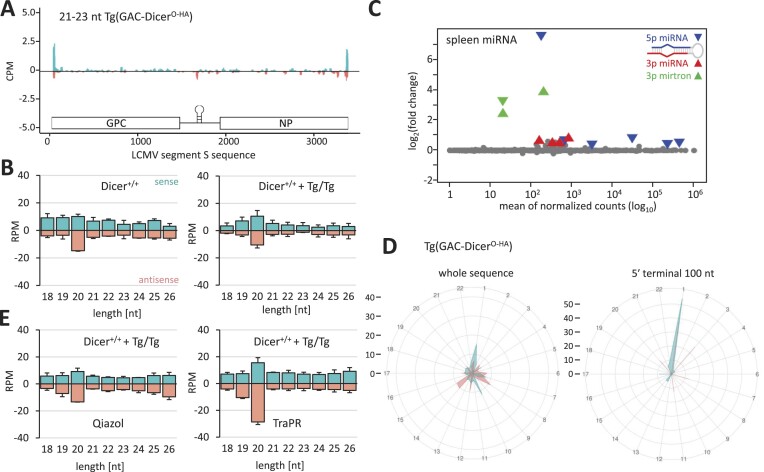
Small RNA analysis in the infected spleens of *Dicer^Tg(O-HA)^* transgenic mice. (**A**) The coverage plot for 21–23-nt small RNAs mapping to the S segment of the LCMV genome depicts combined data obtained from two different spleens at 3 dpi. Animals were infected with 2.5 × 10^6^ PFU of LCMV Arm intraperitoneally. (**B**) Analysis of the length distribution of small RNAs derived from viral RNA in the spleens of infected *Dicer^+/+^+ ^Tg/Tg^* and wild type (Dicer^+/+^) littermates (*n* = 2; error bar = range of values). (**C**) MA plot of differentially expressed miRNAs in *Dicer^Tg(O-HA)^* LCMV-infected spleens relative to wild type infected spleens. (**D**) Phasing analysis of 21–23-nt RNAs from the infected *Dicer^+/+^ +^Tg/Tg^* spleen mapping onto the LCMV sequence. The left diagram is based on analysis of all mapped 21–23-nt RNAs while the right one took into account only those mapping to 100 nt at the RNA termini. (**E**) Length distribution analysis of small RNAs derived from viral RNA in the spleens of infected *Dicer^+/+^+ ^Tg/Tg^* using either normal RNA isolation (Qiazol) or the TraPR approach (*n* = 2; error bar = range of values).

Notably, this antiviral effect was not accompanied by a marked increase in 21–23-nt RNA levels in the spleens of *Dicer^Tg(O-HA)^* homozygous mice relative to *Dicer^wt/wt^* mice (Figure 11A and B). In fact, the 22-nt RNA peak was not apparent in the LCMV-derived small RNA distribution from the spleens. LCMV 21–23-nt RNA in testes and liver were below 1 RPM and considered too low for analysis (data not shown). The distribution of small RNAs was dominated by the 20-nt RNA species (Figure 11B), which was also largely derived from 5′ terminal sequences of mRNAs (Supplementary Figure S3). At the same time, the spleens of LCMV-infected *Dicer^Tg(O-HA)^* animals showed only a small number of differentially expressed miRNAs, which included increased levels of mirtrons (Figure 11C) suggesting the antiviral effect is not associated with a major suppression of Dicer activity in the *Dicer^Tg(O-HA)^* spleens. Phasing analysis of LCMV-derived 21–23-nt RNAs showed the same picture as infection of *Dicer^Δ HEL1/wt^*, i.e. signal in the register 1, which became much stronger when the analysis was restricted to the 5′ terminal 100 nt (Figure 11D), which is consistent with the high 21–23-nt RNA signal at viral RNA termini (Figure 11A). To examine RISC association, we used again the TraPR approach to analyzed small RNAs from infected spleens. Remarkably, we did not observe strong enrichment of 21–23-nt species but the 20-nt RNA species was enriched ∼2-fold (Figure 11E), suggesting that the 20-nt RNA species actually might be loaded onto the RISC.

## Discussion

Our results provide a new framework for antiviral RNAi and bring several important implications. We have genetically engineered the *Dicer^Δ HEL1^* allele in mice to establish an *in vivo* model with enhanced RNAi, which could fill a gap in the research on the antiviral potential of mammalian RNAi. The gap stemmed from the fact that Dicer and AGO2 are essential for the miRNA pathway, so it is impossible to study antiviral RNAi *in vivo* in their absence; this includes the loss of catalytic activity of AGO2 (10,75,76). The *Dicer^Δ HEL1/wt^* mouse offers an experimental alternative where heterozygosity leaves the canonical miRNA pathway essentially intact while the mouse exhibits an order of magnitude higher siRNA production and more efficient RNAi ubiquitously (43).

We analyzed four viruses whose selection primarily stemmed from their availability in collaborating laboratories and established procedures for infecting mice *in vivo*. The viruses were used for testing whether the enhanced RNAi in *Dicer^Δ HEL1/wt^* mutants would be sufficient to provide detectable antiviral effects. In fact, it was previously reported that EMCV is targeted by RNAi in ESCs (26), TBEV is targeted by RNAi in the tick host (45) and that other *Picornaviridae* and *Flaviviridae* viruses are targeted by RNAi in mammals (29,30,32–35). However, *Dicer^Δ HEL1/wt^* mice did not show any reproducible enhanced defense against the tested viruses. We observed suppression of CVB3 in the first experiment, which was not reproduced in subsequent three independent infections. We cannot exclude that the genetic background could play a role as shown before (77). In contrast to EMCV, TBEV and LCMV experiments done in an inbred C57Bl/6 background, CVB3 experiments were performed using the CD1 outbred genetic background onto which was initially crossed the *Dicer^Δ HEL1/wt^* allele, which was prepared in R1 ESCs (129 background). In the very first CVB3 experiment, wild type littermates developed systemic infection while *Dicer^Δ HEL1/wt^* mice did not (Figure 1B). In subsequent experiments, systemic infection did not develop in any animals. We speculate that the CD1 background with a residual 129 background could have affected the outcome. In any case, this initial CVB3 result is an example of experimental variability that could not be reproduced.

RNAi requires efficient processing of long dsRNA into siRNAs, which is not provided by the mammalian full-length Dicer. The N-terminal helicase is the key structural element of Dicer, which facilitates miRNA biogenesis and prevents efficient long dsRNA conversion into siRNA (5,8,12,17). Accordingly, Dicer variants with a modified N-terminal helicase have been examined in mammalian antiviral RNAi studies. Among them stands out the so-called N1 variant lacking HEL1 and HEL2i domains (31,66,78). The N1 variant has increased siRNA biogenesis while it does not interact with dsRNA binding proteins that modulate the Dicer function (78,79). It was also shown in HEK293 cells that the N1 Dicer has antiviral activity against SINV, SFV and EV71 (but not against VSV or SARS-CoV-2), which involves RNAi-independent stimulation of interferon and inflammatory response pathways (31). Another reported endogenous Dicer isoform is the antiviral Dicer (AviD) isoform. It was identified by PCR and lacks the HEL2i domain through skipping exons 7 and 8 during splicing (80). AviD was shown to support antiviral RNAi in HEK293 cells against SINV and ZIKV and it was proposed to provide antiviral protection in intestinal stem cells (80). The physiological role of AviD is unknown but it is apparently non-essential in healthy animals as suggested by phenotype analysis of *Dicer^SOM/SOM^* mice, which lack AviD and could be used for testing the significance of the antiviral role of AviD *in vivo* (43,81).

The Dicer^ΔHEL1^ protein variant used in our study is an HA-tagged functional equivalent of the Dicer^O^ variant. The Dicer^ΔHEL1^ isoform has been structurally and functionally thoroughly investigated (6,13,43). It lacks the HEL1 subdomain, which causes higher siRNA production, but retains HEL2i, which is important for protein–protein interaction (82). Since the *Dicer^Δ HEL1^* mutation preserved the endogenous transcription control of Dicer, *Dicer^Δ HEL1/wt^* mice represent the minimal possible intervention to stimulate siRNA production *in vivo*. However, we observed little if any vsiRNA production and no antiviral effects for CVB3, EMCV, TBEV and LCMV in the *Dicer^Δ HEL1/wt^* mice.

Based on the observed putative siRNA levels, we hypothesized that a single *Dicer^Δ HEL1^* allele may not be sufficient to produce the Dicer activity necessary for antiviral effects. To test whether a further increased Dicer activity could have any measurable effect, we further examined LCMV infections in *Dicer^Δ HEL1/ Δ HEL1^* ESCs and, since *Dicer^Δ HEL1/ Δ HEL1^* animals are not viable, we produced a transgenic model with higher Dicer activity where Dicer^O^ was expressed on top of the normal endogenous Dicer. Indeed, we detected antiviral effects in both cases supporting the idea that antiviral RNAi has a higher Dicer activity threshold than can be delivered by a single *Dicer^Δ HEL1^* allele *in vivo*, but this threshold can be reached with ectopic expression of a truncated Dicer as was the case of the Dicer^O-3^ ESC line and Dicer^Tg(O-HA)^ transgene in LCMV infections.

Apart from Dicer, the amount and accessibility of viral long dsRNA are likely significant limitations for generating enough vsiRNAs for efficient RNAi. vsiRNA RPM values in *Dicer^Δ HEL1/ Δ HEL1^* and *Dicer^O-3^* ESCs were relatively low when compared with high siRNA levels from expressed long dsRNA (Figure 9) and siRNA levels correlating with RNAi silencing in cultured cells or *in vivo* (13,17,43). However, canonical RNAi represents a steady-state system of independently expressed long dsRNA and its complementary target, which is degraded via RISC and thus requires a high amount of AGO-loaded siRNAs. Antiviral RNAi operates on a replicating system where even a minor repression would have a leverage effect over the replication cycles, and the repression may involve both cleavage of the replicating virus by Dicer and targeting viral transcripts by AGO-bound vsiRNAs. Yet, it is still puzzling that a 21–23-nt peak in RNA-seq of LCMV-infected samples was observed in ESCs but not *in vivo*, despite the fact that we observed a reduced amount of LCMV RNA in the *Dicer^Tg(O-HA)^* mice. This contrasted with ESC experiments where vsiRNAs were readily visible as a 21–23-nt peak in the *Dicer^Δ HEL1/ Δ HEL1^* line (Figure 7D). Several factors may contribute to this discrepancy. First, the antiviral effect may occur without accumulating vsiRNAs, as it may also come from the Dicer-mediated cleavage of viral RNA and not just from the AGO2-mediated cleavage required for canonical RNAi. Furthermore, target-mediated decay (52,83) might promote stronger depletion of AGO2-bound vsiRNAs in organs.

While most experiments were performed in animals that are competent in the interferon response, TBEV and LCMV were also tested in the *Pkr* knock-out background to examine whether elimination of this dsRNA sensor could have a positive effect on vsiRNA production. However, in contrast to transient transfections of dsRNA-expressing plasmids (13,66), the loss of Pkr did not stimulate vsiRNA production and RNAi *in vivo*. In the future, it could prove interesting to test mutants of other interferon pathway factor, such as Stat1 (84) or other factors, some of which were shown to enhance RNAi. These include RIG-I (16), Lgp2 (15) or Mavs (85).

Taken together, our research on mice expressing Dicer^ΔHEL1^ variants does not contradict antiviral RNAi in mammals but reframes it and highlights several important considerations for future research. It is important to recognize that mammalian endogenous RNAi is not absent but mostly ineffective because of low Dicer activity, limiting dsRNA substrate levels, and activity of sequence-independent dsRNA responses.

Conversely, presence of high Dicer activity, accessible dsRNA substrate and reduction of sequence-independent dsRNA responses facilitate RNAi. It is thus possible to find experimental conditions to show the RNAi functionality, especially in experimental systems tolerating high Dicer expression, controlling the dsRNA abundance and with reduced interference from sequence-independent dsRNA responses. RNAi may be a negligible nuisance for most viruses but some may be sensitive to it. Future research should further delineate the actual physiological potential of endogenous antiviral RNAi *in vivo* and the utility of transient activation of RNAi via a truncated Dicer variant to provide an additional layer of innate immunity.

## Supplementary Material

gkae1288_Supplemental_File

## Data Availability

The data underlying this article are available in the article and in its online supplementary material. RNA-seq data (Supplementary Table S2) were deposited in the Gene Expression Omnibus database under accession number GSE273338.

## Notes

Present address: Brigita Benköova, Faculty of Medicine, Viral Hepatitis Laboratory, Institute of Microbiology, Slovak Medical University, Limbova 12, 83303 Bratislava, Slovakia.

Present address: Ales Drobek, Faculty of Biology and Medicine, Department of Immunobiology, University of Lausanne, Ch. des Boveresses 155, CH-1066 Epalinges, Switzerland.

Present address: Filip Horvat, Laboratory of DNA Damage Response and Transcription Regulation, Department of Chromatin, RNA and Chromosome Biology, Max Perutz Labs, Vienna Biocenter Campus (VBC), Dr.-Bohr-Gasse 9, 1030, Vienna, Austria.
