# Supplementary material for: Enhanced RNAi does not provide efficient innate antiviral immunity in mice
Source: Nucleic Acids Res. 2025 Jan 9;53(1):gkae1288. doi: 10.1093/nar/gkae1288 (PMC11707545; doi:10.1093/nar/gkae1288)
Supplement: gkae1288_Supplemental_File [file gkae1288_supplemental_file.pdf]

SUPPLEMENTARY MATERIAL

Title:

Enhanced RNAi does not provide efficient innate antiviral immunity in mice.

Authors:

Marcos Iuri Roos Kulmann[1], Eliska Taborska[1], Brigita Benköova[2], Martin Palus[3,4], Ales Drobek[5], Filip Horvat[1,6], Josef Pasulka[1], Radek Malik[1], Eva Salyova[5], Vaclav Hönig[3,4], Michaela Pellerova[2], Maria Borsanyiova[2], Lenka Nedvedova[3,7], Ondrej Stepanek[5], Shubhada Bopegamage[2], Daniel Ruzek[3,4,8], and Petr Svoboda[1+]

Affiliations:

[1] Laboratory of Epigenetic Regulations, Institute of Molecular Genetics of the Czech Academy of Sciences, Videnska 1083, 142 20 Prague 4, Czech Republic

[2] Faculty of Medicine, Enterovirus Laboratory, Institute of Microbiology, Slovak Medical University, Limbova 12, 83303 Bratislava, Slovakia

[3] Institute of Parasitology, Biology Centre of the Czech Academy of Sciences, Branisovska 31, CZ-37005 Ceske Budejovice, Czech Republic.

[4] Laboratory of Emerging Viral Infections, Veterinary Research Institute, Hudcova 70, CZ-62100 Brno, Czech Republic

[5] Laboratory of Adaptive Immunity, Institute of Molecular Genetics of the Czech Academy of Sciences, Videnska 1083, 142 20 Prague 4, Czech Republic

[6] Bioinformatics Group, Division of Molecular Biology, Department of Biology, Faculty of Science, University of Zagreb, 10000, Zagreb, Croatia

[7] Faculty of Science, University of South Bohemia, Branisovska 1645/31a, CZ-37005 Ceske Budejovice, Czech Republic

[8] Department of Experimental Biology, Faculty of Science, Masaryk University, Kamenice 5, CZ-62500 Brno, Czech Republic

[+] correspondence to: Petr Svoboda, Institute of Molecular Genetics of the Czech Academy of Sciences, Videnska 1083, 142 20 Prague 4, Czech Republic, tel. # +420 241063147, e-mail: svobodap@img.cas.cz.

Supplementary Figures and Tables

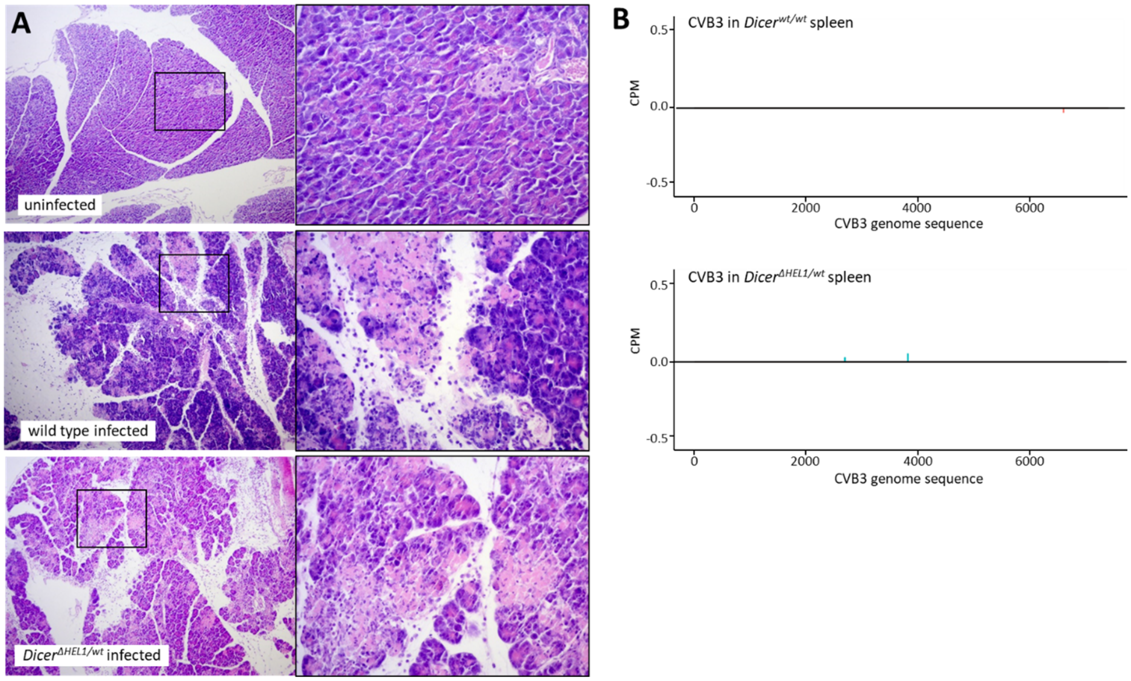

Figure S1 Analysis of small RNAs in mice infected with CVB3. (A) Histological evidence of pancreas tissue infection with CVB3 after intraperitoneal inoculation of wild type (wt) and Dicer $^{\Delta HEL1/wt}$ as compared to sham infected control mice at 3 dpi. Hematoxylin-eosin (HE) staining showed control tissue without infiltration at 3 dpi, the infected wild type tissue showed infiltration and acute inflammation of the pancreatic acinar tissue at 3 dpi. (B) The coverage plot for 21-23 nt small RNAs mapping to the CVB3 genome sequence shows absence of 21-23 nt putative vsiRNA in the spleens of infected mice at 3 dpi. The *Dicer*$^{\Delta HEL1/wt}$ panel is the same as in Fig. 1E for easier comparison with the wild type coverage plot.

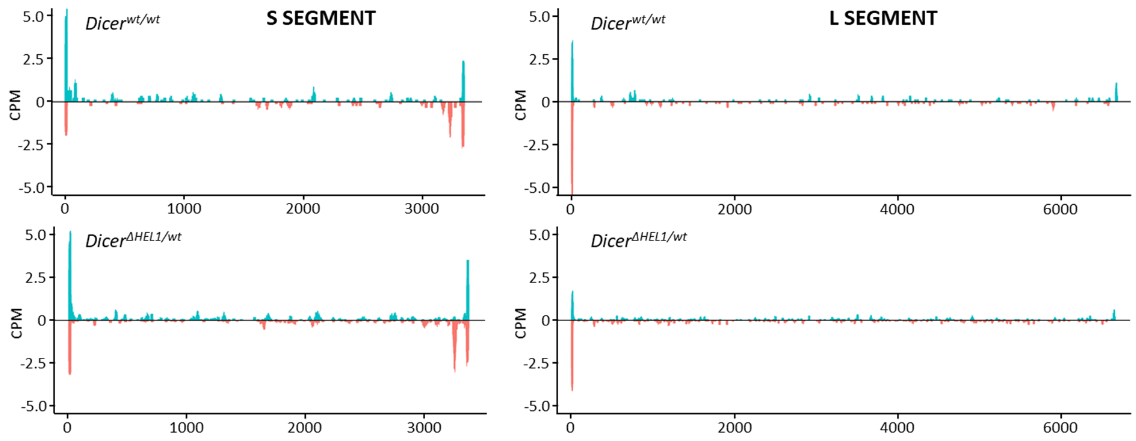

Figure S2 LCMV-derived small RNA analysis in the spleen. Coverage plots showing 21-23 nt reads from the infected spleen mapped onto S and L segments of the LCMV genome.

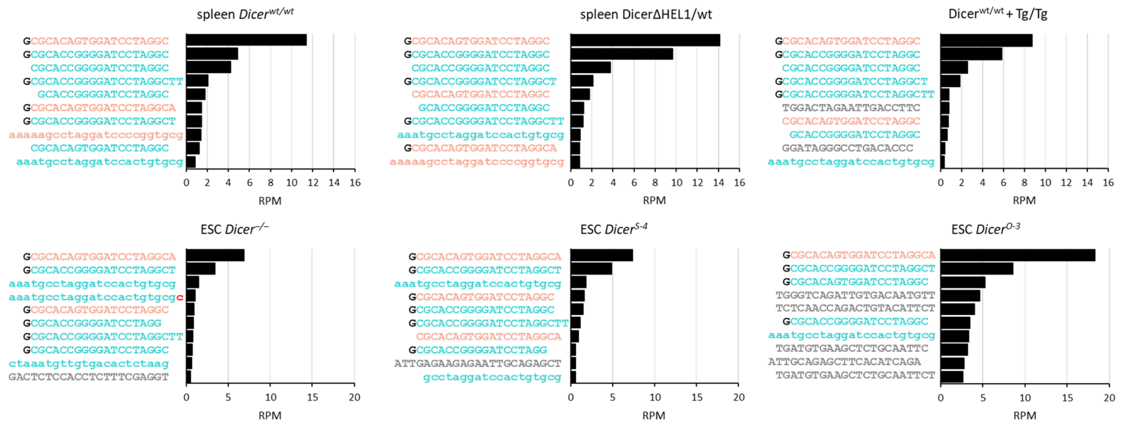

Figure S3 Most abundant 18-24 nt RNAs in depicted small RNA sequencing libraries. Iris blue and salmon fonts indicate RNAs derived from the termini of the LCMV S segment in sense and antisense direction, respectively. Sequences in lower case are complementary to the sequences in upper case. Other LCMV sequences are in grey font. The black 5′ terminal G indicates non-templated G addition in mRNAs. Each sample/genotype was analyzed in a duplicate.

Table S1 Primers

Name	5′ – 3′ sequence	note
mDcr_E4_Fwd	CAAGTGTCAGCCGTCAGAACTCAC	genotyping
mDcr_int4_gen_Rev4	AGGCTGAGGGCTATTAAATCAAGG	genotyping
mDcr_E5_gen_Rev	CGATAGGGGTGGTCTAGAATCGC	genotyping
mPKR_i1_Fwd1	GATATAACCAGCTCAAGTGTTTGC	genotyping
mPKR_E2_gen_Rev	GAGCAAAAAGTTCATCAGGAACC	genotyping
mPKR_E6_Rev1	GCCTGGTTGGGTATAGACTGCTTG	genotyping
mDcr_i1_Fwd2	CAGAGGGCTAGAGCATACAAACAC	genotyping
mDcr_int7_R2	CCCTCGAATTTTGGGACTAACTGC	genotyping
mDcr_i1_Fwd_seq	GAGGACTACACCATCGTGGAACAG	genotyping
mDcr_int7_Rev3	CGTGGTAAGTGGCTTTATACACC	genotyping
mDcr_E6_fwd	GACCACTACCAGCAGAACACCC	genotyping
mRosa26_Fwd1	CACGTTTCCGACTTGAGTTGCCTC	genotyping
mRosa26_Rev1	GATAAGGCTGCAGAAGGAGCGG	genotyping
mCherry_qPCR_Fwd1	TCAAGCAGAGGCTGAAGCTGAA	genotyping
mRosa26_i1_F2	AGGCAGGAAGCACTTGCTCTCC	genotyping
mRosa26i1_R	GGAAAATACTCCGAGGCGGATCAC	genotyping
LCMV_Fwd	CGCTGGCCTGGGTGAATTG	qPCR
LCMV_Rev	GTGAAGGATGGCCATACATAGC	qPCR
EMCV_Fwd	GACGCTTGAAGACGTTGTCTTCTTA	qPCR
EMCV_Rev	CCCTACCTCACGGAATGGGGCAAAG	qPCR
mHGPRT.1_457_Fwd	GCTACTGTAATGATCAGTCAACGG	qPCR
mHGPRT.1_670_Rev	CTGTATCCAACACTTCGAGAGGTC	qPCR
mB2MG.1_342_Fwd	GCAGAGTTAAGCATGCCAGTATGG	qPCR
mB2MG.1_514_Rev	CATTGCTATTTCTTTCTGCGTGC	qPCR
mALAS.1_1274_Fwd	CACTGGAAGAGCTGTGTGACGTG	qPCR
mALAS.1_1463_Rev	CTGGCAATGTATCCTCCAACACAG	qPCR
eEF1a1_Fwd	ACACGTAGATTCCGGCAAGT	qPCR
eEF1a1_Fwd	AGGAGCCCTTTCCCATCTC	qPCR

Table S2 Sequencing libraries deposited in GEO

genotype	GEO library name	note	virus	age	organ
Dicer$^{wt/wt}$	spleen_WT_CVB3_B25S	Fig. 1E, 1F & S1B	CVB3	juvenile (4 weeks)	spleen
Dicer$^{wt/wt}$	spleen_WT_CVB3_S33	Fig. 1E, 1F & S1B	CVB3	juvenile (4 weeks)	spleen
Dicer$^{\Delta HEL1/wt}$	spleen_DicerDHEL1het_CVB3_S26	Fig. 1E, 1F & S1B	CVB3	juvenile (4 weeks)	spleen
Dicer$^{\Delta HEL1/wt}$	spleen_DicerDHEL1het_CVB3_S25	Fig. 1E, 1F & S1B	CVB3	juvenile (4 weeks)	spleen
Dicer$^{wt/wt}$	heart_WT_EMCV_46900	Fig. 2B-E	EMCV	young adult (6 weeks)	heart
Dicer$^{wt/wt}$	heart_WT_EMCV_46906	Fig. 2B-E	EMCV	young adult (6 weeks)	heart
Dicer$^{\Delta HEL1/wt}$	heart_DHEL1het_EMCV_46908	Fig. 2B-E	EMCV	young adult (6 weeks)	heart
Dicer$^{\Delta HEL1/wt}$	heart_DHEL1het_EMCV_46907	Fig. 2B-E	EMCV	young adult (6 weeks)	heart
Dicer$^{wt/wt}$	brain_WT_TBEV_4	Fig. 3D-G	TBEV	young adult (6-7 weeks)	brain
Dicer$^{wt/wt}$	brain_WT_TBEV_5	Fig. 3D-G	TBEV	young adult (6-7 weeks)	brain
Dicer$^{wt/wt}$	brain_WT_TBEV_6	Fig. 3D-G	TBEV	young adult (6-7 weeks)	brain
Dicer$^{\Delta HEL1/wt}$	brain_DicerDHEL1het_TBEV_1	Fig. 3D-G	TBEV	young adult (6-7 weeks)	brain
Dicer$^{\Delta HEL1/wt}$	brain_DicerDHEL1het_TBEV_2	Fig. 3D-G	TBEV	young adult (6-7 weeks)	brain
Dicer$^{\Delta HEL1/wt}$	brain_DicerDHEL1het_TBEV_3	Fig. 3D-G	TBEV	young adult (6-7 weeks)	brain
Dicer$^{wt/wt}$, *Pkr*$^{-/-}$	brain_PKRnull_TBEV_3dpi	Fig. 4D, 4E	TBEV	young adult (6-7 weeks)	brain
Dicer$^{wt/wt}$, *Pkr*$^{-/-}$	brain_PKRnull_TBEV_6dpi	Fig. 4D, 4E	TBEV	young adult (6-7 weeks)	brain
Dicer$^{wt/wt}$, *Pkr*$^{-/-}$	brain_PKRnull_TBEV_10dpi	Fig. 4D, 4E	TBEV	young adult (6-7 weeks)	brain
Dicer$^{\Delta HEL1/wt}$, *Pkr*$^{-/-}$	brain_DicerDHEL1het_PKRnull_TBEV_3dpi	Fig. 4D, 4E	TBEV	young adult (6-7 weeks)	brain
Dicer$^{\Delta HEL1/wt}$, *Pkr*$^{-/-}$	brain_DicerDHEL1het_PKRnull_TBEV_6dpi	Fig. 4D, 4E	TBEV	young adult (6-7 weeks)	brain
Dicer$^{\Delta HEL1/wt}$, *Pkr*$^{-/-}$	brain_DicerDHEL1het_PKRnull_TBEV_10dpi	Fig. 4D, 4E	TBEV	young adult (6-7 weeks)	brain
Dicer$^{wt/wt}$	spleen_WT_LCMV_28Y35226	Fig. 5B, 5C, 11B	LCMV	adult (12 weeks)	spleen
Dicer$^{wt/wt}$	spleen_WT_LCMV_28Y35286	Fig. 5B, 5C, 11B	LCMV	adult (12 weeks)	spleen
Dicer$^{\Delta HEL1/wt}$	spleen_DicerDHEL1het_LCMV_28Y35225	Fig. 5B,5C, 5F, 6C, 6D	LCMV	adult (12 weeks)	spleen
Dicer$^{\Delta HEL1/wt}$	spleen_DicerDHEL1het_LCMV_28Y35291	Fig. 5B,5C, 5F, 6C, 6D	LCMV	adult (12 weeks)	spleen
Dicer$^{tg(O-HA)/tg(O-HA)}$	spleen_DicerTG_LCMV_28Y35336	Fig. 11A-C	LCMV	adult (12 weeks)	spleen
Dicer$^{tg(O-HA)/tg(O-HA)}$	spleen_DicerTG_LCMV_28Y35338	Fig. 11A-C	LCMV	adult (12 weeks)	spleen
Dicer$^{wt/wt}$, *Pkr*$^{-/-}$	spleen_PKRnull_LCMV_3dpi	Fig.5D, 5E	LCMV	adult (12 weeks)	spleen
Dicer$^{\Delta HEL1/wt}$, *Pkr*$^{-/-}$	spleen_DicerDHEL1het_PKRnull_LCMV_3dpi	Fig.5D, 5E	LCMV	adult (12 weeks)	spleen
Dicer$^{wt/wt}$	mESC_WT_LCMV_MOI_0.01_7	Fig. 7C, 7D	LCMV	cell line	ESC
Dicer$^{wt/wt}$	mESC_WT_LCMV_MOI_0.01_8	Fig. 7C, 7D	LCMV	cell line	ESC
Dicer$^{\Delta HEL1/\Delta HEL1}$	mESC_DicerDHEL1_LCMV_MOI_0.01_9	Fig. 7C, 7D	LCMV	cell line	ESC
Dicer$^{\Delta HEL1/\Delta HEL1}$	mESC_DicerDHEL1_LCMV_MOI_0.01_10	Fig. 7C, 7D	LCMV	cell line	ESC
Dicer$^{\Delta HEL1/\Delta HEL1}$, *Pkr*$^{-/-}$	mESC_DicerDHEL1_PKRnull_LCMV_MOI_0.01_11	Fig. 7C, 7D	LCMV	cell line	ESC
Dicer$^{\Delta HEL1/\Delta HEL1}$, *Pkr*$^{-/-}$	mESC_DicerDHEL1_PKRnull_LCMV_MOI_0.01_12	Fig. 7C, 7D	LCMV	cell line	ESC
Dicer$^{wt/wt}$	mESC_WT_LCMV_MOI_1_15	Fig. 7E	LCMV	cell line	ESC
Dicer$^{wt/wt}$	mESC_WT_LCMV_MOI_1_16	Fig. 7E	LCMV	cell line	ESC
Dicer$^{\Delta HEL1/\Delta HEL1}$	mESC_DicerDHEL1_LCMV_MOI_1_19	Fig. 7E	LCMV	cell line	ESC
Dicer$^{\Delta HEL1/\Delta HEL1}$	mESC_DicerDHEL1_LCMV_MOI_1_20	Fig. 7E	LCMV	cell line	ESC
Dicer$^{\Delta HEL1/\Delta HEL1}$, *Pkr*$^{-/-}$	mESC_DicerDHEL1_PKRnull_LCMV_MOI_1_23	Fig. 7E	LCMV	cell line	ESC
Dicer$^{\Delta HEL1/\Delta HEL1}$, *Pkr*$^{-/-}$	mESC_DicerDHEL1_PKRnull_LCMV_MOI_1_24	Fig. 7E	LCMV	cell line	ESC
Dicer$^{-/-}$	mESC_DicerKO_LCMV_MOI_0.01_1	Fig. 8B	LCMV	cell line	ESC
Dicer$^{-/-}$	mESC_DicerKO_LCMV_MOI_0.01_2	Fig. 8B	LCMV	cell line	ESC
Dicer^{O-3}	mESC_DicerO3_LCMV_MOI_0.01_1	Fig. 8B-D	LCMV	cell line	ESC
Dicer^{O-3}	mESC_DicerO3_LCMV_MOI_0.01_2	Fig. 8B-D	LCMV	cell line	ESC
Dicer^{S-4}	mESC_DicerS4_LCMV_MOI_0.01_1	Fig. 8B	LCMV	cell line	ESC
Dicer^{S-4}	mESC_DicerS4_LCMV_MOI_0.01_2	Fig. 8B	LCMV	cell line	ESC
Dicer$^{wt/wt}$	mESC_WT_LCMV_MOI_0.01_S9	Fig. 9A	LCMV	cell line	ESC
Dicer$^{wt/wt}$	mESC_WT_LCMV_MOI_0.01_S10	Fig. 9A	LCMV	cell line	ESC
Dicer$^{\Delta HEL1/\Delta HEL1}$	mESC_DicerDHEL1_LCMV_MOI_0.01_S11	Fig. 9A	LCMV	cell line	ESC
Dicer$^{\Delta HEL1/\Delta HEL1}$	mESC_DicerDHEL1_LCMV_MOI_0.01_S12	Fig. 9A	LCMV	cell line	ESC
Dicer^{O-3}	mESC_DicerO3_LCMV_MOI_0.01_S15	Fig. 9A	LCMV	cell line	ESC
Dicer^{O-3}	mESC_DicerO3_LCMV_MOI_0.01_S16	Fig. 9A	LCMV	cell line	ESC
Dicer$^{wt/wt}$	mESC_WT_MosIR_S1	Fig. 9B	MosIR	cell line	ESC
Dicer$^{wt/wt}$	mESC_WT_MosIR_S2	Fig. 9B	MosIR	cell line	ESC
Dicer$^{\Delta HEL1/\Delta HEL1}$	mESC_DicerDHEL1_MosIR_S3	Fig. 9B	MosIR	cell line	ESC
Dicer$^{\Delta HEL1/\Delta HEL1}$	mESC_DicerDHEL1_MosIR_S4	Fig. 9B	MosIR	cell line	ESC
Dicer^{O-3}	mESC_Dicer03_MosIR_S7	Fig. 9B	MosIR	cell line	ESC
Dicer^{O-3}	mESC_Dicer03_MosIR_S8	Fig. 9B	MosIR	cell line	ESC
Dicer$^{wt/wt}$	mESC_WT_LCMV_MOI_0.01_TrapR_S9	Fig. 9A	LCMV	cell line	ESC
Dicer$^{wt/wt}$	mESC_WT_LCMV_MOI_0.01_TrapR_S10	Fig. 9A	LCMV	cell line	ESC
Dicer$^{\Delta HEL1/\Delta HEL1}$	mESC_DicerDHEL1_LCMV_MOI_0.01_TrapR_S11	Fig. 9A	LCMV	cell line	ESC
Dicer$^{\Delta HEL1/\Delta HEL1}$	mESC_DicerDHEL1_LCMV_MOI_0.01_TrapR_S12	Fig. 9A	LCMV	cell line	ESC
Dicer^{O-3}	mESC_Dicer03_LCMV_MOI_0.01_TrapR_S15	Fig. 9A	LCMV	cell line	ESC
Dicer^{O-3}	mESC_Dicer03_LCMV_MOI_0.01_TrapR_S16	Fig. 9A	LCMV	cell line	ESC
Dicer$^{wt/wt}$	mESC_WT_MosIR_TrapR_S1	Fig. 9B	MosIR	cell line	ESC
Dicer$^{wt/wt}$	mESC_WT_MosIR_TrapR_S2	Fig. 9B	MosIR	cell line	ESC
Dicer$^{\Delta HEL1/\Delta HEL1}$	mESC_DicerDHEL1_MosIR_TrapR_S3	Fig. 9B	MosIR	cell line	ESC
Dicer$^{\Delta HEL1/\Delta HEL1}$	mESC_DicerDHEL1_MosIR_TrapR_S4	Fig. 9B	MosIR	cell line	ESC
Dicer^{O-3}	mESC_Dicer03_MosIR_TrapR_S7	Fig. 9B	MosIR	cell line	ESC
Dicer^{O-3}	mESC_Dicer03_MosIR_TrapR_S8	Fig. 9B	MosIR	cell line	ESC
Dicer$^{wt/wt}$	spleen_WT_LCMV_Qiazol_50069	Fig. 11E	LCMV	adult (11 weeks)	spleen
Dicer$^{wt/wt}$	spleen_WT_LCMV_Qiazol_50070	Fig. 11E	LCMV	adult (11 weeks)	spleen
Dicer$^{tg(O-HA)/tg(O-HA)}$	spleen_DicerTG_LCMV_Qiazol_50041	Fig. 11D, 11E	LCMV	adult (11 weeks)	spleen
Dicer$^{tg(O-HA)/tg(O-HA)}$	spleen_DicerTG_LCMV_Qiazol_50302	Fig. 11D, 11E	LCMV	adult (11 weeks)	spleen
Dicer$^{wt/wt}$	spleen_WT_LCMV_TraPR_50069	Fig. 11E	LCMV	adult (11 weeks)	spleen
Dicer$^{wt/wt}$	spleen_WT_LCMV_TraPR_50070	Fig. 11E	LCMV	adult (11 weeks)	spleen
Dicer$^{tg(O-HA)/tg(O-HA)}$	spleen_DicerTG_LCMV_TraPR_50041	Fig. 11D, 11E	LCMV	adult (11 weeks)	spleen
Dicer$^{tg(O-HA)/tg(O-HA)}$	spleen_DicerTG_LCMV_TraPR_50302	Fig. 11D, 11E	LCMV	adult (11 weeks)	spleen
